# A Multi-Strategy Kangaroo Escape Optimization Technique for Global Optimization, Engineering Design, and Near-Infrared Prediction of Praeruptorin Content

**DOI:** 10.3390/biomimetics11080587

**Published:** 2026-08-17

**Authors:** Jingya Zhang, Yu Liu, Chaochuan Jia, Maosheng Fu, Xinyu Gao, Yubao Zhu, Qiqi Zhang

**Affiliations:** 1 School of Electronic Information and Artificial Intelligence, West Anhui University, Lu’an 237012, China; 2 Anhui Province Intelligent Hydraulic Machinery Joint Construction Subject Key Laboratory, Lu’an 237012, China

**Keywords:** Kangaroo Escape Optimization Technique, metaheuristic optimization, differential evolution, opposition-based learning, engineering design, near-infrared spectroscopy, backpropagation neural network

## Abstract

The Kangaroo Escape Optimization Technique (KET) combines escape and safe-area searches, but its fixed stage allocation, restricted guidance range, limited refinement of low-ranked individuals, and insufficient use of population-state information can reduce its performance on complex problems. This study develops a Multi-Strategy Kangaroo Escape Optimization Technique (MSKET) through iteration-dependent stage switching, population-proportion-based candidate guidance, selective greedy DE/rand-to-best/1 refinement, and beta-distribution opposition-based global-best guidance. The contribution lies in assigning established mechanisms to specific KET limitations and coordinating them across different stages and population subsets. MSKET was evaluated through 30 independent runs on the 100-dimensional CEC2017 and CEC2020 suites. It ranked first on 22 of 29 CEC2017 functions and 8 of 10 CEC2020 functions, with the best average rank on both suites. Ablation, diversity, and nonparametric statistical analyses further supported the observed performance gains. MSKET also achieved the best overall repeated-run results on the piston–lever and three-bar truss design problems. For near-infrared prediction, MSKET-BP obtained an R^2^ of 0.86440, an RMSE of 2.1377, and a MAPE of 5.0083% on the testing set. These results indicate improved search and prediction performance within the examined tasks, although at a higher computational cost than KET.

## 1. Introduction

Optimization problems in engineering design, model calibration, parameter identification, and data-driven prediction are often nonlinear, multimodal, constrained, and high-dimensional. In such settings, gradient information may be unavailable or expensive to obtain, while strong variable interactions can limit conventional deterministic methods [1,2,3]. Metaheuristic algorithms provide a derivative-free alternative through population-based search. Representative methods include Particle Swarm Optimization (PSO), Harris Hawks Optimization (HHO), the Slime Mould Algorithm (SMA), and the Newton Downhill Optimizer (NDO), which employ different information-sharing and position-update mechanisms [4,5,6,7]. Despite their different formulations, no single algorithm performs consistently well across all problem classes. Rapid diversity loss may cause premature convergence, whereas insufficient local refinement can limit final solution accuracy. These weaknesses become more evident on high-dimensional and complex multimodal landscapes. Current research therefore increasingly focuses on improving existing search structures and coordinating global exploration with local exploitation.

Recent variants of metaheuristic algorithms have used strategy selection, adaptive grouping, opposition-based learning, and stage-specific updates to address weaknesses in their base optimizers. The effect of each mechanism depends on its role, activation stage, and target individuals. Table 1 compares four representative methods published between 2022 and 2025 with MSKET, with emphasis on mechanism placement rather than the number of added operators [8,9,10,11].

These studies indicate that established mechanisms are more useful when their roles within the base algorithm are clearly defined. MSEFA selects among alternative search strategies, AGSMA separates population roles, ABetaCOBL adapts the opposition domain, and ISO assigns different mechanisms to successive search phases. The original KET lacks comparable role-specific control. MSKET addresses this issue by linking four mechanisms to four identified weaknesses and activating them by search stage or population subset. The individual mechanisms are established; the methodological contribution lies in their KET-specific coordination.

Metaheuristic and data-driven methods are increasingly used in engineering tasks that combine numerical modelling, experimental data, and performance prediction. Shafaie and Movahedi Rad integrated a multi-objective genetic algorithm with PFC3D to calibrate discrete-element parameters for ten coloured self-compacting concrete mixtures, illustrating the use of evolutionary search in multi-objective model calibration [12]. Shafaie et al. subsequently combined push-out testing with fuzzy logic to predict the shear bond strength of fibre-reinforced self-compacting concrete, achieving R^2^ values of up to 0.96 [13]. These studies show that intelligent computation can support both physics-based parameter calibration and data-driven property estimation. Such applications also require reliable performance across constrained design and prediction tasks. Accordingly, this study evaluates MSKET on engineering design problems and near-infrared prediction rather than relying solely on benchmark functions.

The Kangaroo Escape Optimization Technique (KET) is a population-based optimizer inspired by kangaroo escape behaviour [14]. Its search process combines long-jump escape, zigzag escape, safe-area search, and decoy-mask updating. Long-jump escape enables large displacements, whereas zigzag escape changes the search direction around the global-best direction. Safe-area search guides individuals toward selected promising regions, and the decoy mask determines which dimensions are updated. Despite this structure, four limitations remain. The stage-selection rule is fixed, the safe-area candidate set is small, low-ranked individuals receive no additional refinement, and the neighbourhood of the global best is not sufficiently explored. These limitations may reduce convergence accuracy and run-to-run stability on complex high-dimensional problems.

Based on these limitations, this study develops MSKET while retaining the original escape-search framework of KET. An iteration-dependent switching rule adjusts the use of escape and safe-area search. A population-proportion-based candidate group broadens the source of safe-area guidance. Low-ranked individuals are then refined by a greedy DE/rand-to-best/1 operator, and beta-distribution opposition-based guidance generates supplementary candidates around promising regions. These mechanisms are assigned to different search stages or population subsets rather than applied uniformly. Adaptive control, differential mutation, population guidance, and opposition-based learning are established concepts; the contribution of MSKET lies in their KET-specific assignment to stage regulation, guidance selection, low-ranked-individual refinement, and global-best information utilization. MSKET is evaluated on the 100-dimensional CEC2017 and CEC2020 suites. The assessment includes ablation experiments, population diversity analysis, function evaluations, CPU time, computational complexity, and Wilcoxon, Friedman, Iman–Davenport, and Nemenyi tests. Two constrained engineering design problems and an MSKET-optimized BP model for near-infrared prediction are also examined.

The main contributions of this study are threefold. First, MSKET restructures the KET search process by assigning adaptive stage switching, candidate-group-based safe-area guidance, selective greedy DE/rand-to-best/1 refinement, and beta-distribution opposition guidance to four identified limitations. The contribution lies in the KET-specific coordination of established mechanisms rather than in proposing new standalone operators. Second, MSKET is assessed on the 100-dimensional CEC2017 and CEC2020 suites through accuracy, convergence, stability, ablation, population diversity, function evaluations, CPU time, computational complexity, and nonparametric statistical tests. Third, its performance beyond benchmark functions is examined using piston–lever and three-bar truss design problems and an MSKET-BP model for near-infrared prediction of total praeruptorin content. Section 2 presents KET and MSKET. Section 3 reports the benchmark experiments and supplementary analyses. Section 4 describes the engineering design and near-infrared prediction applications. Section 5 concludes the study.

## 2. Proposed Methodology

### 2.1. Original Kangaroo Escape Optimization Technique

The Kangaroo Escape Optimization Technique (KET) is a swarm intelligence optimizer based on kangaroo escape behaviour [14]. In KET, each individual represents a candidate solution, and the population is updated through long-jump escape, zigzag escape, safe-area search, and decoy-mask operations.

At the beginning of the search, KET randomly initializes the population and evaluates the fitness of each individual. The best individual found so far is used as the main guide. Long-jump escape supports large movements across the search space, while zigzag escape changes the movement direction by combining the current position, a randomly selected individual, and the global best solution. Safe-area search guides individuals toward safer or better regions, and the decoy-mask mechanism controls the updated dimensions.

Although KET combines escape and safe-area searches, four limitations remain in its update process. The stage-selection rule does not adapt to search progress, the safe-area guide is chosen from a restricted candidate range, low-ranked individuals receive no secondary refinement after the main update, and population-state information is not used to generate supplementary candidates for the global best. These limitations define the four modification targets of MSKET.

### 2.2. Multi-Strategy Kangaroo Escape Optimization Technique

MSKET retains the original search framework of KET and modifies four parts of its update process. An adaptive stage-switching rule regulates the use of escape and safe-area searches, while a population-proportion-based candidate group expands the source of safe-area guidance. DE/rand-to-best/1 and beta-distribution opposition-based learning are established mechanisms [15,16]. In MSKET, they are applied selectively to the refinement of low-ranked individuals and the generation of supplementary candidates for global-best updating, respectively. The distinction of MSKET lies in assigning these mechanisms to specific KET limitations and executing them at different stages or on different population subsets.

Throughout this section, *N* and *D* denote the population size and problem dimension, respectively. The position of individual *i* at iteration *t* is represented by $x_{i}^{t}$, while $x_{\mathrm{best}}^{t}$ and $F_{\mathrm{best}}^{t}$ denote the current best solution and its fitness value. The vectors *lb* and *ub* define the search bounds, and f(⋅) is the objective function. The random terms satisfy ρ~U(0,1), $\xi \sim U{\left(0,1\right)}^{D}$, γ~N(0,1), and $g\sim N(0,I_{D})$.

#### 2.2.1. Population Initialization

MSKET follows the initialization rule of the original KET [14]. Each coordinate is sampled independently within its feasible interval according to Equation (1):

(1) $$x_{i,j}^{0}=lb_{j}+\rho_{i,j}(ub_{j}-lb_{j}),\;i=1,2,\ldots ,N,\;j=1,2,\ldots ,D.$$

where $\rho_{i,j}\sim U(0,1)$, and $lb_{j}$ and $ub_{j}$ are the lower and upper bounds of the j-th variable, respectively. The initialized population is evaluated using f(⋅), and the best solution is stored as $x_{\mathrm{best}}^{0}$.

#### 2.2.2. Adaptive Stage Switching and Energy Regulation Mechanism

To replace the non-adaptive stage allocation of KET, MSKET defines an iteration-dependent probability for selecting Stage 1. The probability at iteration *t* is given by Equation (2).

(2) $$p_{s}^{t}=0.8-0.5\frac{t}{T}$$

where $p_{s}^{t}$ denotes the probability of selecting Stage 1 at iteration *t*. For each individual, an independent random scalar $\rho_{s,i}^{t}\sim U(0,1)$ is generated. If $\rho_{s,i}^{t}<p_{s}^{t}$, the individual enters Stage 1 and performs escape search; otherwise, it enters Stage 2 and performs safe-area search. As *t* increases from 1 to *T*, $p_{s}^{t}$ decreases from approximately 0.8 to 0.3. Escape search is therefore selected more often in the early iterations, whereas safe-area search becomes more frequent later. This rule changes the allocation of the two KET stages without altering their respective update operators.

After entering Stage 1, the individual further selects long-jump escape or zigzag escape according to the energy level. The energy level of the *i*-th individual at iteration *t* is calculated as shown in Equation (3).

(3) $$E_{i}^{t}=\left(1-\rho_{e,i}^{t}\frac{t}{T}\right)\left(0.95+0.05\chi^{t}\right)$$

where $\rho_{e,i}^{t}\sim U(0,1)$, $\chi^{t}$ denotes the logistic chaotic factor, and $E_{i}^{t}$ is the energy level of individual *i* at iteration *t*. The chaotic factor is initialized as $\chi^{1}=0.7$ and updated by $\chi^{t}=4\chi^{t-1}(1-\chi^{t-1})$. Equation (3) combines iteration-dependent random attenuation with bounded chaotic modulation. Within Stage 1, long-jump escape is selected only when $E_{i}^{t}>0.5$ and the stochastic trigger condition is satisfied; otherwise, zigzag escape is used. The energy level therefore determines the choice between the two retained KET updates and does not directly modify the individual position.

#### 2.2.3. Long-Jump Escape and Zigzag Escape Update

MSKET retains the long-jump and zigzag escape updates of the original KET as the two branches of Stage 1 [14]. Their selection is determined by the energy level and stochastic trigger defined in Section 2.2.2. When long-jump escape is selected, a binary dimensional mask $m_{i}^{t}=\mathrm{round}(\xi_{i}^{t})$ is generated, where $\xi_{i}^{t}\sim U{\left(0,1\right)}^{D}$. If all elements of the mask are zero, one randomly selected element is set to 1. The resulting candidate is defined by Equation (4).

(4) $$v_{i}^{t}=x_{i}^{t}+2\gamma_{i}^{t}\left(m_{i}^{t}⊙x_{i}^{t}\right),\;\sum_{j=1}^{D}m_{i,j}^{t}\geq 1$$

where $v_{i}^{t}$ denotes the candidate solution generated by long-jump escape, $\gamma_{i}^{t}\sim N(0,1)$ is a Gaussian random scalar, $m_{i}^{t}\in {\left\{0,1\right\}}^{D}$ is the binary dimensional mask, and ⊙ denotes element-wise multiplication. Equation (4) perturbs only the selected dimensions and supports large-step search in partial dimensions.

If long-jump escape is not selected, zigzag escape is adopted. This update combines the direction from the current individual to the global best individual with an orthogonal perturbation direction. The zigzag escape update is shown in Equation (5).

(5) $$\begin{array}{l} \theta_{i}^{t}=\theta_{\max}\left(2\rho_{\theta ,i}^{t}-1\right)\\ d_{i}^{t}=x_{i}^{t}-x_{\mathrm{best}}^{t}\\ q_{i}^{t}=\cos(\theta_{i}^{t})d_{i}^{t}+\sin(\theta_{i}^{t})h_{i}^{t}\|d_{i}^{t}{\|}_{2}\\ v_{i}^{t}=x_{i}^{t}+\beta_{z}\mathrm{sign}(\theta_{i}^{t})\left(g_{i}^{t}⊙q_{i}^{t}\right) \end{array}$$

where $\theta_{i}^{t}$ is the random deflection angle, $\rho_{\theta ,i}^{t}\sim U(0,1)$, and $\theta_{\max}=30{}^{\circ}$. The vector $d_{i}^{t}$ points from the current individual toward the global-best solution. The unit vector $h_{i}^{t}$ is obtained by orthogonalizing and normalizing a random vector with respect to $d_{i}^{t}$, such that $\langle h_{i}^{t},d_{i}^{t}\rangle =0$. The vector $q_{i}^{t}$ denotes the rotated zigzag direction, and $\|d_{i}^{t}{\|}_{2}$ is the Euclidean norm of $d_{i}^{t}$. Moreover, $g_{i}^{t}\sim N(0,I_{D})$ is a *D*-dimensional Gaussian random vector, ⊙ denotes element-wise multiplication, and $\beta_{z}$ is the zigzag perturbation coefficient, set to 1 in this study. Equation (5) combines angular rotation of the global-best displacement direction with dimension-wise Gaussian perturbation to avoid purely straight-line movement.

#### 2.2.4. Safe-Area Search and Candidate-Group Guidance Mechanism

Stage 2 retains the decoy-mask rule and safe-area position update of KET but changes how the guiding individual is selected [14]. The decoy mask is first generated by Equation (6).

(6) $$m_{i}^{t}=\left\{\begin{array}{ll} 1_{D}, & \rho_{m,i}^{t}<1/3\\ \mathrm{round}(\xi_{1,i}^{t}), & 1/3\leq \rho_{m,i}^{t}<2/3\\ \mathrm{round}(\xi_{1,i}^{t}⊙\xi_{2,i}^{t}), & \rho_{m,i}^{t}\geq 2/3 \end{array}\right.$$

where $\rho_{m,i}^{t}\sim U(0,1)$ and $\xi_{1,i}^{t},\xi_{2,i}^{t}\sim U{\left(0,1\right)}^{D}$. Here, $1_{D}$ denotes a *D*-dimensional all-one vector. Equation (6) generates masks with different sparsity levels, allowing the safe-area search to update all dimensions or only part of the dimensions.

Let $s_{i}^{t}$ denote the safe-area guiding individual for individual *i*. During the first half of the iterations, or under an independent stochastic condition with probability 0.5, the guide is sampled uniformly from the current population. Otherwise, the guide is selected from the candidate group with probability 0.75 or set to the current global best with a probability of 0.25. The safe-area search update is given in Equation (7).

(7) $$v_{i}^{t}=s_{i}^{t}+\gamma_{i}^{t}m_{i}^{t}⊙(x_{i}^{t}-s_{i}^{t})$$

where $v_{i}^{t}$ denotes the candidate solution generated by safe-area search, and $\gamma_{i}^{t}\sim N(0,1)$ is a Gaussian random scalar. Equation (7) constructs the search step based on the difference between the current individual and the safe-area guiding individual, while the decoy mask controls the updated dimensions.

To broaden the selection range of safe-area guiding individuals, MSKET constructs a candidate group according to the population size. The candidate group size is defined as shown in Equation (8).

(8) $$K_{s}=\max(1,\mathrm{round}(0.30N))$$

where $K_{s}$ denotes the number of individuals in the candidate group. The candidate-group size scales with the population size and replaces the restricted guide range used in the original KET. This modification changes guide selection only; the safe-area position update remains defined by Equation (7). After generating the candidate solution, boundary handling is applied, and a greedy selection rule is used to retain the better individual.

#### 2.2.5. Greedy DE/Rand-to-Best/1 Mutation Mechanism for Low-Ranked Individuals

DE/rand-to-best/1 is an established differential mutation strategy [15]. In MSKET, it is applied selectively rather than to the entire population. After the main KET update, all individuals are ranked in ascending order of fitness. Only individuals satisfying $\mathrm{rank}(i)\geq \left\lceil 0.7N\right\rceil$ enter the secondary refinement step, where rank(i) denotes the fitness rank of individual *i* in the current population.

For each selected individual, MSKET generates a DE/rand-to-best/1 candidate solution by Equation (9).

(9) $$u_{i}^{t}=\left\{\begin{array}{ll} x_{r1}^{t}+F_{i}^{t}(x_{\mathrm{best}}^{t}-x_{r1}^{t})+F_{i}^{t}(x_{r2}^{t}-x_{r3}^{t}), & \rho_{d,i}^{t}>0.5\\ x_{i}^{t}, & \rho_{d,i}^{t}\leq 0.5 \end{array}\right.$$

where $u_{i}^{t}$ denotes the generated mutation candidate. $F_{i}^{t}\sim U(0,1)$ is the differential scaling factor, and $\rho_{d,i}^{t}\sim U(0,1)$ determines whether the mutation step is performed. $x_{r1}^{t}$, $x_{r2}^{t}$, and $x_{r3}^{t}$ are three individuals sampled from the current population. The mutation combines global-best guidance with differential variation among three sampled individuals.

After boundary handling, the mutation candidate replaces the current individual only if it yields a lower objective value. The DE component therefore provides selective post-update refinement for low-ranked individuals without perturbing higher-ranked solutions.

#### 2.2.6. Beta-Distribution Opposition-Based Global-Best Guidance Enhancement Mechanism

Beta-distribution opposition-based learning with an adaptive search space is used to generate supplementary candidates for global-best updating [10,16]. Let ${\tilde{x}}_{i}^{t}$ denote individual *i* after the main KET update, boundary handling, DE refinement, and greedy selection. The dynamic population bounds in each dimension are defined by Equation (10).

(10) $$d_{\max,j}^{t}=\max_{1\leq i\leq N}{\tilde{x}}_{i,j}^{t},\;d_{\min,j}^{t}=\min_{1\leq i\leq N}{\tilde{x}}_{i,j}^{t},\;j=1,2,\ldots ,D$$

where $d_{\max,j}^{t}$ and $d_{\min,j}^{t}$ denote the maximum and minimum values of the current population in the *j*-th dimension, respectively. Compared with fixed boundaries, these dynamic boundaries better describe the current search region.

To measure the population distribution, the normalized nearest-neighbour distance of the *i*-th individual is calculated as shown in Equation (11).

(11) $$CD_{i}^{t}=\min_{j\neq i}\frac{{\left\|\left({\tilde{x}}_{i}^{t}-{\tilde{x}}_{j}^{t})⊘(d_{\max^{t}}-d_{\min^{t}}\right)\right\|}_{2}}{\sqrt{D}}$$

where $CD_{i}^{t}$ denotes the nearest-neighbour crowding distance of the *i*-th individual, ⊘ denotes element-wise division, and $\|\cdot {\|}_{2}$ denotes the Euclidean norm. The average crowding distance of the population is then obtained by Equation (12).

(12) $${\bar{CD}}^{t}=\frac{1}{N}\sum_{i=1}^{N}CD_{i}^{t}$$

where ${\bar{CD}}^{t}$ reflects the overall dispersion of the current population. A smaller value indicates a more concentrated population, whereas a larger value indicates a more dispersed population.

Based on ${\bar{CD}}^{t}$, the spread factor of the beta distribution is determined as shown in Equation (13).

(13) $$s^{t}=\left\{\begin{array}{ll} {\left(1/\sqrt{{\bar{CD}}^{t}}\right)}^{1+\eta^{t}}, & \rho_{b}^{t}<0.5\\ 0.1\sqrt{{\bar{CD}}^{t}}+0.9, & \rho_{b}^{t}\geq 0.5 \end{array}\right.$$

where $s^{t}$ is the spread factor of the beta distribution, $\rho_{b}^{t}\sim U(0,1)$, and $\eta^{t}\sim N(0,0.5^{2})$. The spread factor is used to regulate the shape parameters of the beta distribution rather than directly updating individual positions.

The normalized mode position is defined as $u_{i,j}^{t}=\left(d_{\max,j}^{t}-{\tilde{x}}_{i,j}^{t}\right)/\left(d_{\max,j}^{t}-d_{\min,j}^{t}\right)$ in the concave mode and $u_{i,j}^{t}=\left({\tilde{x}}_{i,j}^{t}-d_{\min,j}^{t}\right)/\left(d_{\max,j}^{t}-d_{\min,j}^{t}\right)$ in the convex mode. When $u_{i,j}^{t}<0.5$, $p_{i,j}^{t}=\left[\left(s^{t}-2\right)u_{i,j}^{t}+1\right]/\left[s^{t}\left(1-u_{i,j}^{t}\right)\right]$, $\alpha_{i,j}^{t}=s^{t}p_{i,j}^{t}$, and $\beta_{i,j}^{t}=s^{t}$. Otherwise, $p_{i,j}^{t}=\left(2-s^{t}\right)/s^{t}+\left(s^{t}-1\right)/\left(s^{t}u_{i,j}^{t}\right)$, $\alpha_{i,j}^{t}=s^{t}$, and $\beta_{i,j}^{t}=s^{t}p_{i,j}^{t}$. These parameters define the beta random vector used in Equation (14).

(14) $$o_{i}^{t}=d_{\min^{t}}+(d_{\max^{t}}-d_{\min^{t}})⊙B_{i}^{t},\;B_{i,j}^{t}\sim \mathrm{Beta}\left(\alpha_{i,j}^{t}(u_{i,j}^{t},s^{t}),\beta_{i,j}^{t}(u_{i,j}^{t},s^{t})\right),\;j=1,2,\ldots ,D$$

where $o_{i}^{t}$ denotes the candidate solution generated by beta-distribution opposition-based learning. $B_{i}^{t}=[B_{i,1}^{t},B_{i,2}^{t},\ldots ,B_{i,D}^{t}]$ is a beta random vector, and $B_{i,j}^{t}$ is the beta random sample in the *j*-th dimension. After boundary handling, each opposition candidate is compared with the current global best. If the candidate yields a lower objective value, $x_{\mathrm{best}}^{t}$ and $F_{\mathrm{best}}^{t}$ are updated; otherwise, the current global best is retained. In the present implementation, the opposition candidate is used only to challenge the global best and does not replace the corresponding population member.

#### 2.2.7. Overall Procedure of MSKET

**Figure 1:**
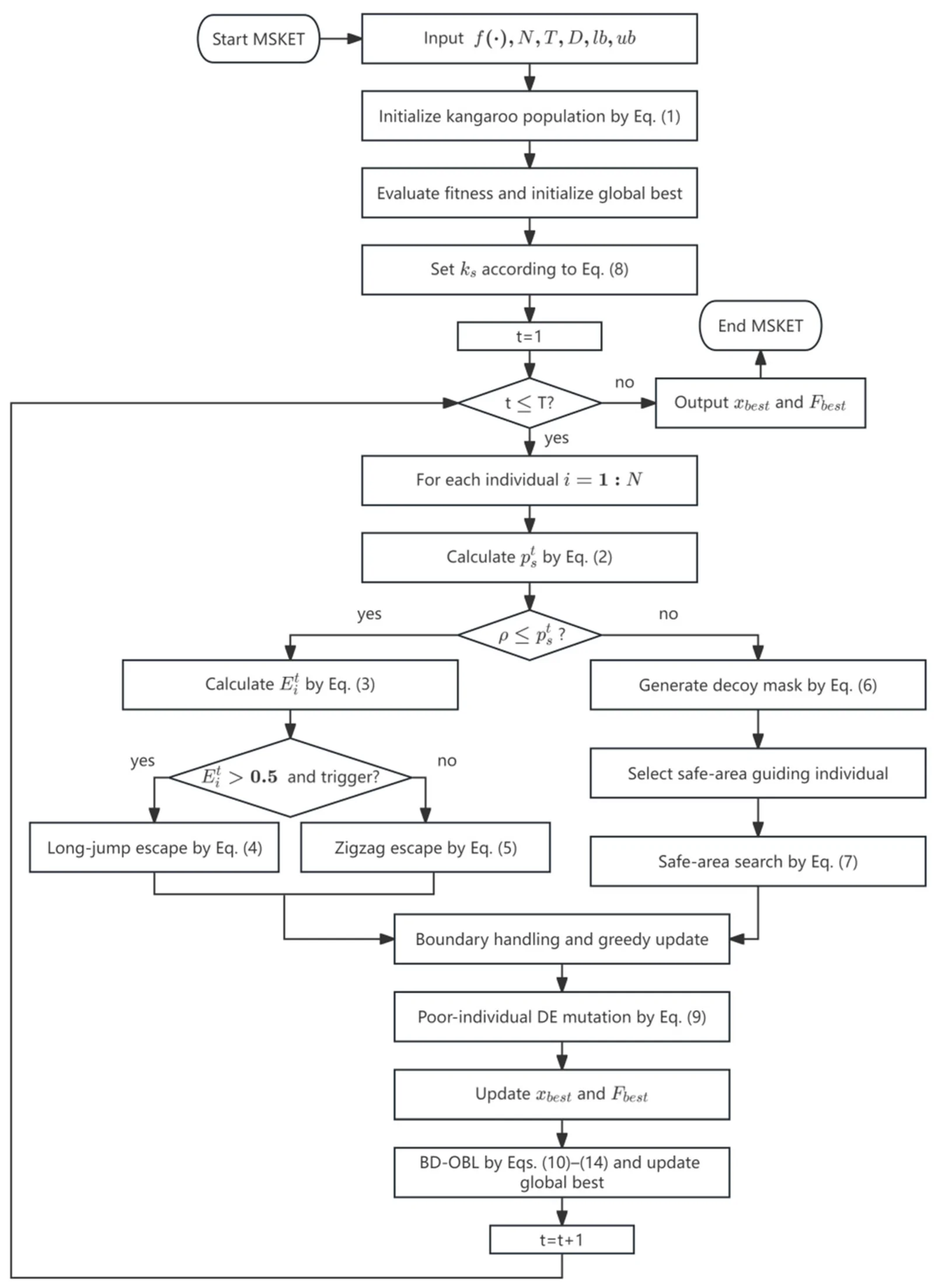
Flowchart of MSKET.

The overall procedure of MSKET is illustrated in Figure 1. The corresponding pseudocode and algorithm-specific parameter settings are presented in Algorithm 1 and Table 2, respectively.
**Algorithm 1.** Main procedure of MSKETBegin1. Input: f(⋅), *N*, *T*, *D*, *lb*, and *ub*.2. Initialize the kangaroo population *X* according to Equation (1).3. Evaluate the fitness values of all individuals and determine the initial global best solution $x_{best}$ and its fitness value $F_{best}$.4. Set the safe-area candidate group size $K_{s}$ according to Equation (8).5. *t* = 16. while *t* ≤ *T* do  7. for *i* = 1, …, *N* do   8. Calculate the Stage 1 execution probability $p_{s}^{t}$ according to Equation (2).   9. if rand < $p_{s}^{t}$ then    10. Calculate the energy level $E_{i}^{t}$ according to Equation (3).    11. if $E_{i}^{t}$ > 0.5 and the random trigger condition is satisfied then     12. Perform long-jump escape update according to Equation (4).    13. else     14. Perform Zigzag escape update according to Equation (5).    15. end if   16. else    17. Generate the decoy mask according to Equation (6).    18. Perform safe-area search update according to Equation (7).   19. end if   20. Apply boundary handling and evaluate the fitness value of the new position.   21. If $f(v_{i}^{t})<f(x_{i}^{t})$, replace $x_{i}^{t}$ with $v_{i}^{t}$.  22. end for  23. Apply greedy DE/rand-to-best/1 refinement to the low-ranked individuals according to Equation (9).  24. Update $x_{best}$ and $F_{best}$ using the accepted population.  25. Perform Beta-distribution OBL according to Equations (10)–(14) and update the global best.  26. set *t* = *t* + 127. end while28. Return $x_{best}$ and $F_{best}$.End MSKET

Within each iteration, adaptive stage selection first determines the main KET update. The accepted population is then ranked, and only the low-ranked subset undergoes DE refinement. The resulting population provides the dynamic bounds and crowding information used by beta-distribution opposition learning, after which the global best is updated. This execution order assigns a distinct role to each mechanism and avoids applying all enhancements uniformly to the population. The search terminates when the maximum number of iterations is reached.

## 3. Experimental Results and Analysis

### 3.1. Experimental Setup

The experiments were conducted on the CEC2017 and CEC2020 benchmark suites [17,18]. MSKET was compared with the original KET and eight algorithms: NDO [7], ESC [19], DBA [20], ALA [21], HHO [5], PSO [4], SMA [6], and UFIA [22].

All methods were coded in MATLAB R2023a. The common configuration used 30 individuals, 1000 iterations, and 100 decision variables. For each function, 30 independent trials were performed with MATLAB’s default random stream, without a fixed seed sequence.

The minimum, mean, standard deviation, and rank were recorded. Rankings were determined by the mean objective value, with the standard deviation used only to resolve ties. The same runs were used to generate the statistical tables, convergence curves, box plots, Wilcoxon tests, average-rank charts, and radar charts.

### 3.2. CEC2017 Test Functions

The 29 CEC2017 functions, with F2 excluded, were used to evaluate high-dimensional optimization performance [17]. The suite includes unimodal, multimodal, hybrid, and composition functions with different levels of variable interaction and landscape complexity.

**Figure 2:**
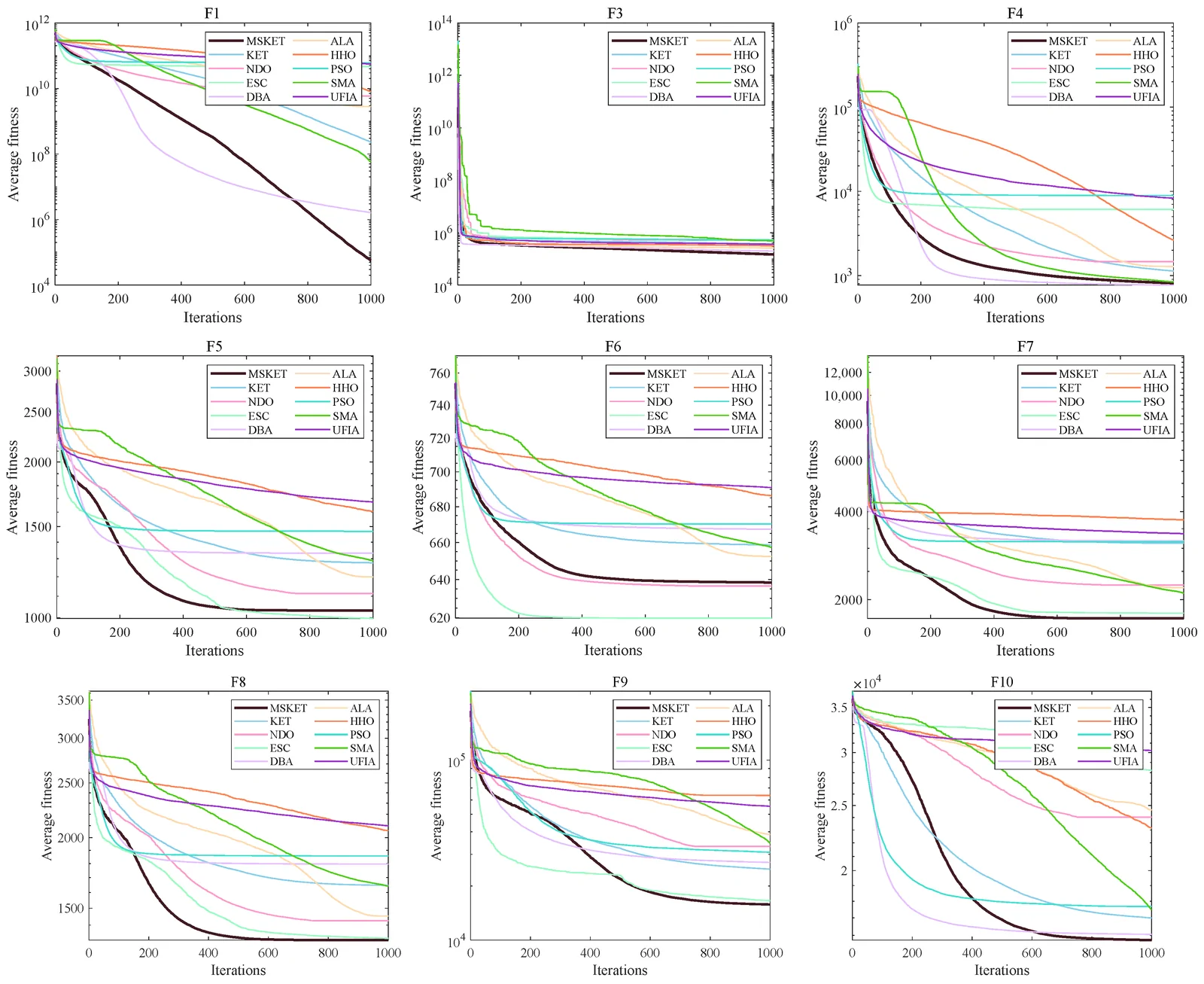
Mean convergence curves for CEC2017 functions F1 and F3–F10.

**Figure 3:**
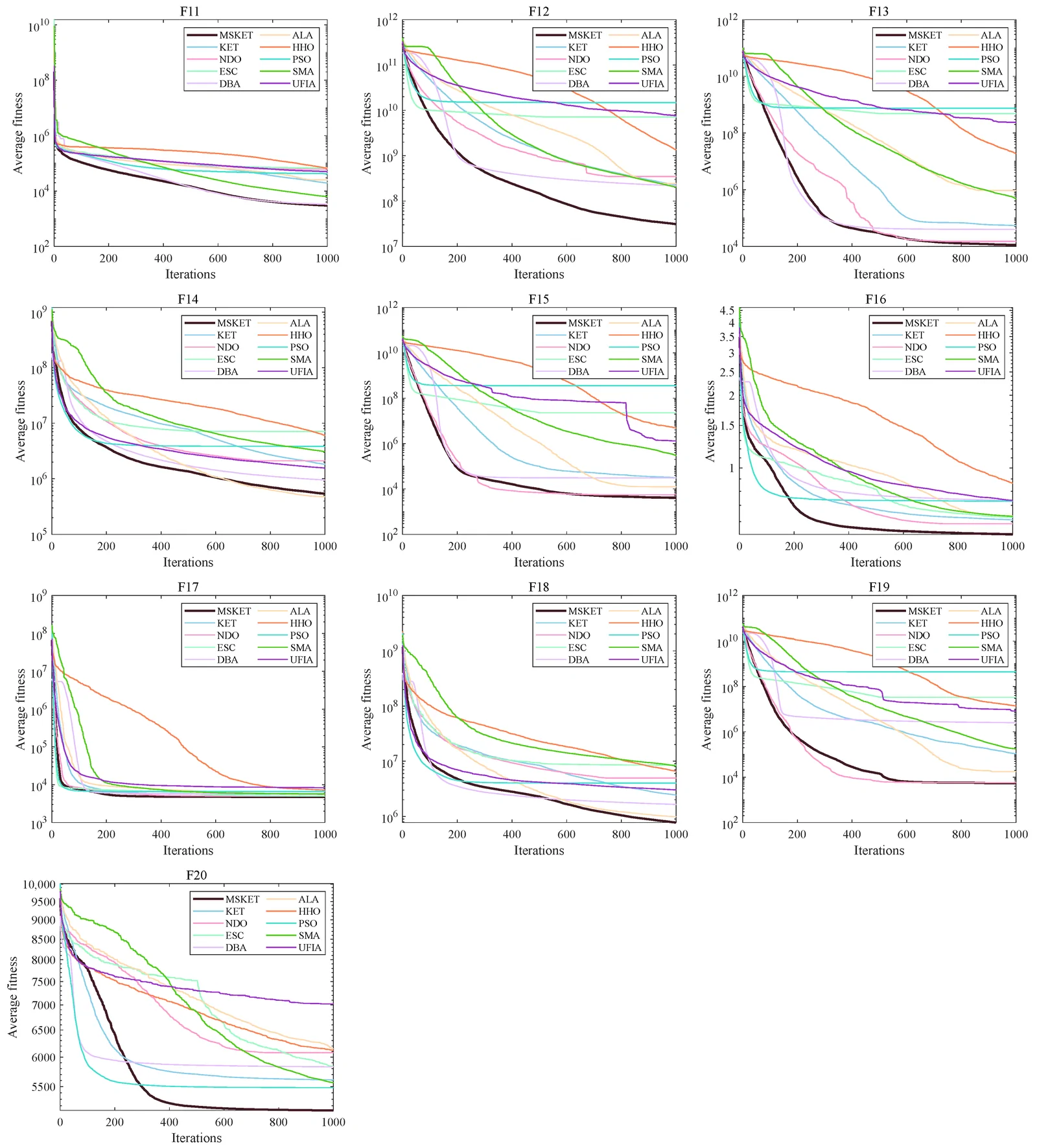
Mean convergence curves for CEC2017 hybrid functions F11–F20.

**Figure 4:**
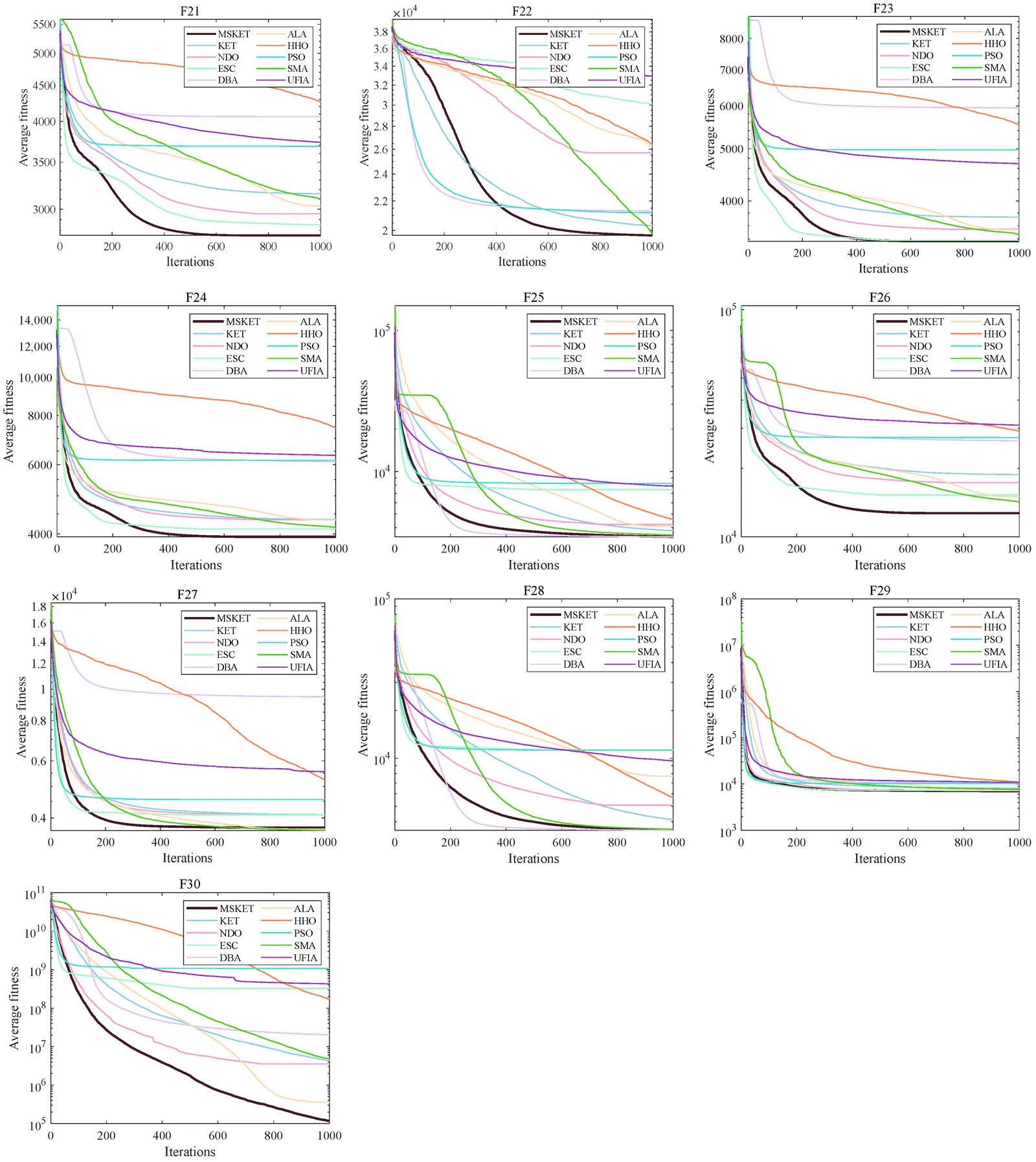
Mean convergence curves for CEC2017 composition functions F21–F30.

**Figure 5:**
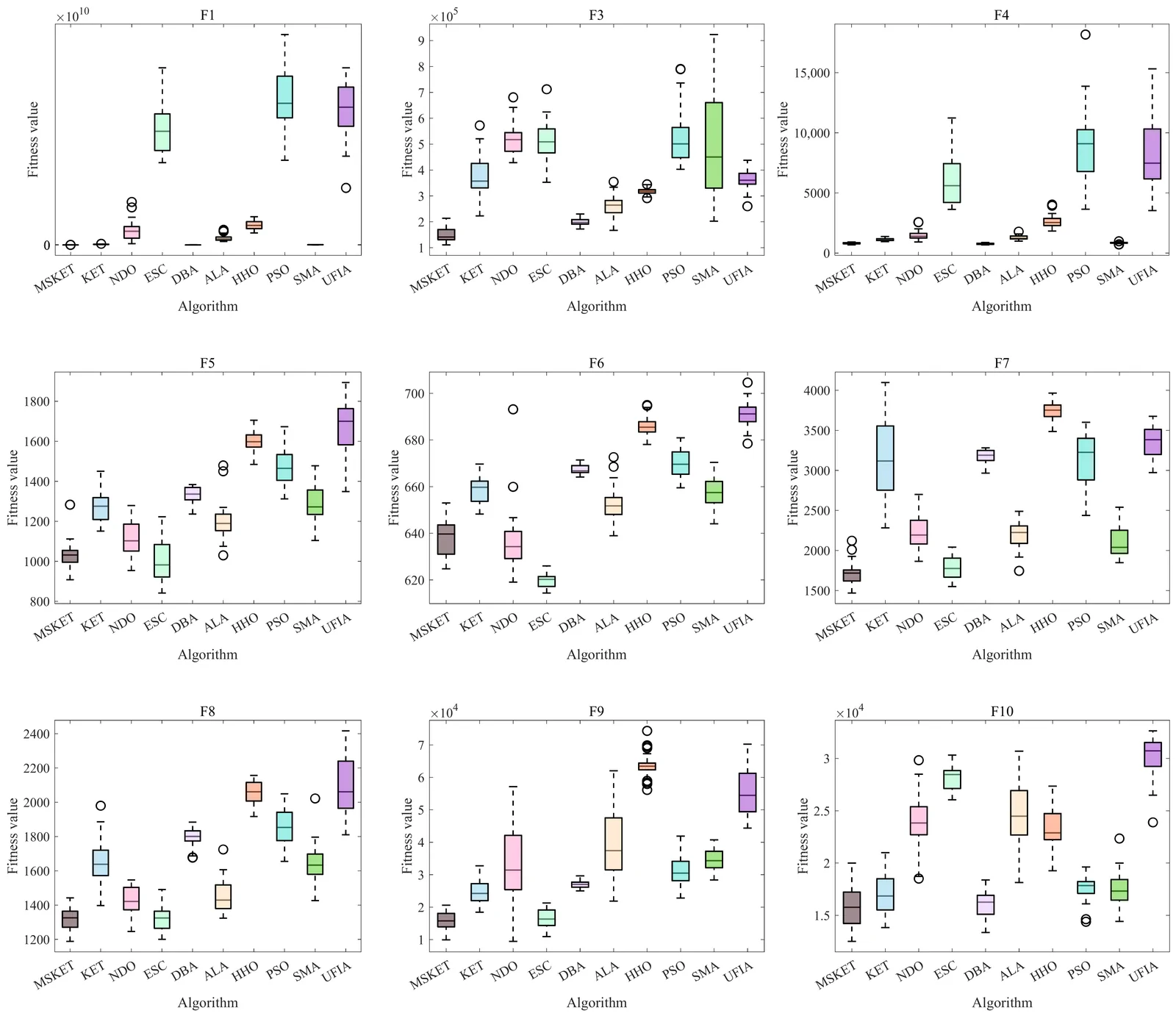
Box plots of CEC2017 test functions F1 and F3–F10. Circles in the box plots indicate outliers.

**Figure 6:**
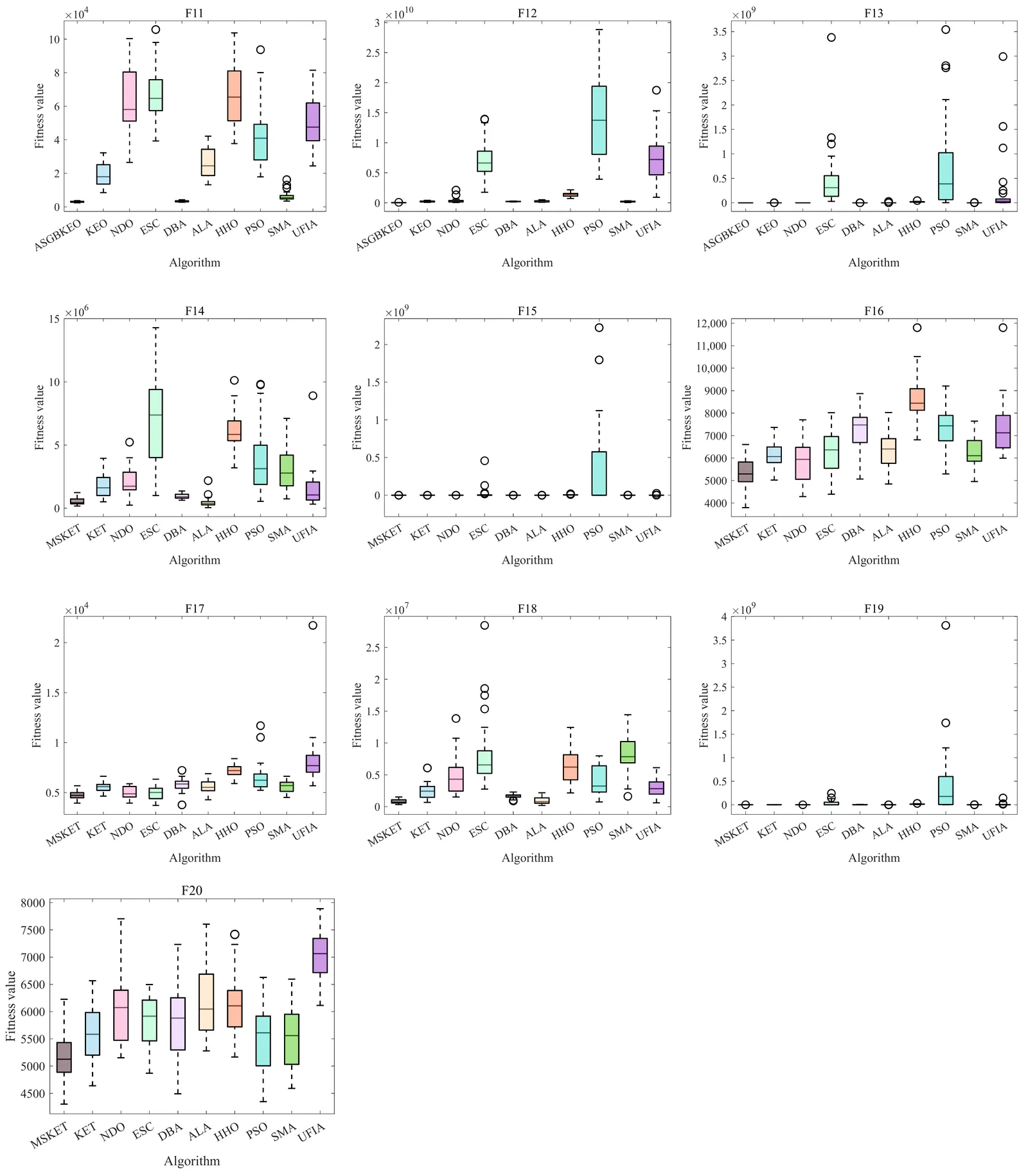
Box plots of CEC2017 test functions F11–F20. Circles in the box plots indicate outliers.

**Figure 7:**
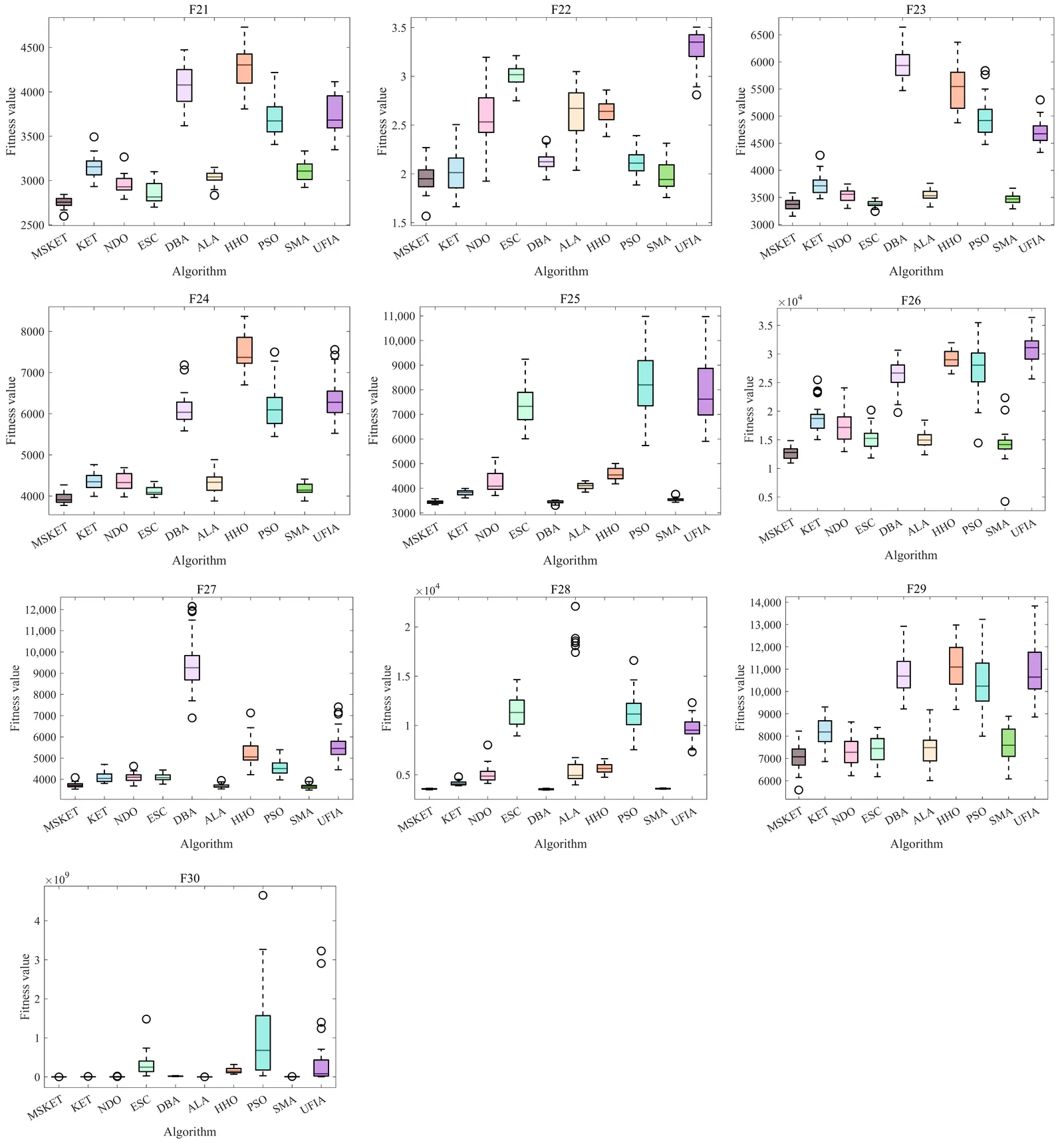
Box plots of CEC2017 test functions F21–F30. Circles in the box plots indicate outliers.

Table 3, Table 4 and Table 5 report the numerical results. Figure 2, Figure 3 and Figure 4 present the convergence trajectories, whereas Figure 5, Figure 6 and Figure 7 show the distributions of the final solutions. Statistical significance and overall performance are examined through the Wilcoxon test, average ranks, and radar charts.

#### 3.2.1. Analysis of Key Indicators for CEC2017

Table 3 reports the results for F1 and F3–F10. MSKET ranked first on F1, F3, and F7–F10, second on F4 and F5, and third on F6. It therefore remained within the top three on all nine functions. On F1 and F3, its mean objective values were 5.66 × 10^4^ and 1.49 × 10^5^, respectively, both lower than those obtained by KET. The results on F7–F10 further show that MSKET retained competitive performance on multimodal landscapes.

The hybrid-function results are listed in Table 4. MSKET ranked first on nine of the ten functions and second on F14. Marked improvements over KET were observed on several functions. For example, the mean value decreased from 2.25 × 10^8^ to 3.11 × 10^7^ on F12, from 3.12 × 10^4^ to 4.07 × 10^3^ on F15, and from 2.44 × 10^6^ to 7.73 × 10^5^ on F18. These results show that the modified search process was effective on functions with stronger variable coupling.

Table 5 presents the composition function results. MSKET ranked first on F21–F24, F26, F29, and F30; second on F25 and F28; and third on F27. Its advantage over KET was particularly pronounced on F30, where the mean value decreased from 4.37 × 10^6^ to 1.17 × 10^5^. Improvements were also obtained on F26 and F29.

Across the 29 CEC2017 functions, MSKET ranked first on 22 functions and remained within the top three on every function. The results in Table 3, Table 4 and Table 5 show consistent performance across unimodal, multimodal, hybrid, and composition landscapes, with the largest gains appearing on several high-dimensional hybrid and composition functions.

#### 3.2.2. Convergence Analysis of CEC2017

Figure 2 presents the mean convergence profiles for F1 and F3–F10. On F1, MSKET continues to reduce the fitness value after the initial decline and reaches the lowest final level. On F3, most algorithms improve rapidly at the beginning, whereas MSKET maintains further progress and obtains the best final result, consistent with Table 3.

For the multimodal functions F4–F10, MSKET achieves the lowest final fitness values on F7–F10. Its curves separate from most competitors during the middle or later iterations, indicating sustained refinement. On F4–F6, MSKET does not rank first, but the convergence trajectories remain smooth and the final values are competitive.

Figure 3 presents the mean convergence curves for the hybrid functions F11–F20. Compared with the functions in Figure 2, these functions involve stronger variable interactions and produce clearer differences among the algorithms. MSKET maintains continued improvement on most functions and generally reaches low final fitness values.

On F12, F13, F15, F16, and F20, MSKET obtains lower final convergence levels than most comparison algorithms. For F12, F13, and F15, which have relatively large fitness scales, MSKET continues to improve after the early rapid decrease and maintains competitive late-stage performance. On F16 and F20, its convergence curves are also smooth and stable.

For F11, F17, F18, and F19, several algorithms converge rapidly in the early stage, and the final gaps are relatively small. Nevertheless, MSKET still reaches low final average fitness values without obvious oscillations. On F14, although MSKET is slightly inferior to the best-performing algorithm, it remains among the leading algorithms. These results show that MSKET has stable convergence behaviour on the F11–F20 hybrid functions.

Figure 4 presents the mean convergence curves for the CEC2017 composition functions F21–F30. These functions have more complex landscapes than the previous groups and impose higher requirements on robustness. Overall, MSKET maintains a steady decrease on most functions and reaches relatively low final fitness values.

On F21–F24, F26, F29, and F30, MSKET obtains lower final fitness levels than most comparison algorithms. On F25 and F28, although MSKET is slightly inferior to the best-performing algorithm, its curves remain stable and its final results are still competitive. On F27, MSKET does not achieve the best final result but remains among the leading algorithms.

These results indicate that MSKET maintains stable convergence behaviour on the CEC2017 composition functions.

Taken together, the convergence curves indicate that MSKET has stable convergence behaviour on the CEC2017 test functions. On most functions, MSKET reduces the fitness value rapidly in the early iterations and continues to refine the solution in the later stages. Compared with the original KET, MSKET reaches lower final fitness values on more functions and shows fewer cases of late-stage stagnation, suggesting better sustained search performance in 100-dimensional search spaces.

#### 3.2.3. Stability Analysis of CEC2017

Figure 5 summarizes the final fitness distributions for F1 and F3–F10 over 30 independent runs. Lower medians indicate better central performance, whereas narrower interquartile ranges and fewer outliers reflect greater run-to-run consistency.

Overall, MSKET obtains low median values and compact distributions on most functions. For the unimodal functions F1 and F3, its boxes are located at relatively low levels with small fluctuations. For the multimodal functions F4–F10, MSKET shows lower medians than most comparison algorithms on F7, F8, F9, and F10. On F5 and F6, although MSKET does not obtain the lowest boxes, its distributions remain compact. On F4, MSKET is close to several well-performing algorithms and maintains a low median value.

These box plot results show that MSKET has stable performance on F1 and F3–F10. Compared with the original KET, MSKET generally produces more concentrated result distributions over 30 independent runs.

Figure 6 shows the box plots of all algorithms on the CEC2017 hybrid functions F11–F20 over 30 independent runs. These functions have more complex structures and stronger variable coupling, so the box plots provide further evidence of algorithmic stability in complex search spaces.

For F11–F20, Figure 6 shows that MSKET achieves comparatively low medians and narrow interquartile ranges on F11–F13, F15, F18, and F19. On F17, the leading algorithms perform similarly, while MSKET retains limited dispersion. Its median is not the lowest on F14, F16, or F20, but the distributions remain competitive. Several comparison methods exhibit broader spreads or more outliers, indicating greater run-to-run variability.

These results are consistent with the statistical indicators and convergence curves, showing that MSKET maintains stable performance on the F11–F20 hybrid functions.

Figure 7 summarizes the final fitness distributions for the composition functions F21–F30. MSKET yields low medians and narrow interquartile ranges on F21–F24, F26, F29, and F30. Its results on F25 and F28 remain close to those of the leading methods. Although the median on F27 is slightly higher than the best result, the dispersion is limited. These distributions indicate consistent repeated-run performance across the composition functions.

Several comparison algorithms present wider box spans or more outliers on F23, F24, F25, F27, and F29. In contrast, MSKET maintains compact result distributions on most F21–F30 functions, which is consistent with the statistical ranking results.

Overall, the box plots show that MSKET produces compact result distributions on different types of CEC2017 functions, with median values generally located at low levels. Compared with the original KET and most comparison algorithms, MSKET shows smaller fluctuations across unimodal, multimodal, hybrid, and composition functions, confirming its stable performance over 30 independent runs.

#### 3.2.4. Statistical Analysis of CEC2017

Pairwise differences between MSKET and each competing algorithm were evaluated using the Wilcoxon rank-sum test [23]. Average ranks across the 29 CEC2017 functions were also computed to summarize overall performance.

Table 6 lists the pairwise Wilcoxon results for the CEC2017 benchmark suite. In this table, “+”, “−”, and “=” denote that MSKET is significantly better than, significantly worse than, or statistically comparable to the corresponding algorithm, respectively. The “+/−/=” summary provides a direct statistical comparison between MSKET and the other algorithms.

As shown in Table 6, MSKET obtains more “+” cases than “−” cases in all pairwise comparisons. It achieves 29/0/0 against HHO, PSO, and UFIA, indicating significant advantages on all 29 CEC2017 functions. Compared with the original KET, MSKET obtains 28/0/1, with no significantly inferior cases.

MSKET also performs favourably against NDO, ALA, SMA, DBA, and ESC. The results against NDO and ALA are both 25/0/4, and no “−” case is observed. Although several “−” cases appear in the comparisons with ESC, DBA, and SMA, the number of “+” cases remains larger. In the average ranking results, MSKET ranks first among all algorithms, which is consistent with the Wilcoxon test results.

**Figure 8:**
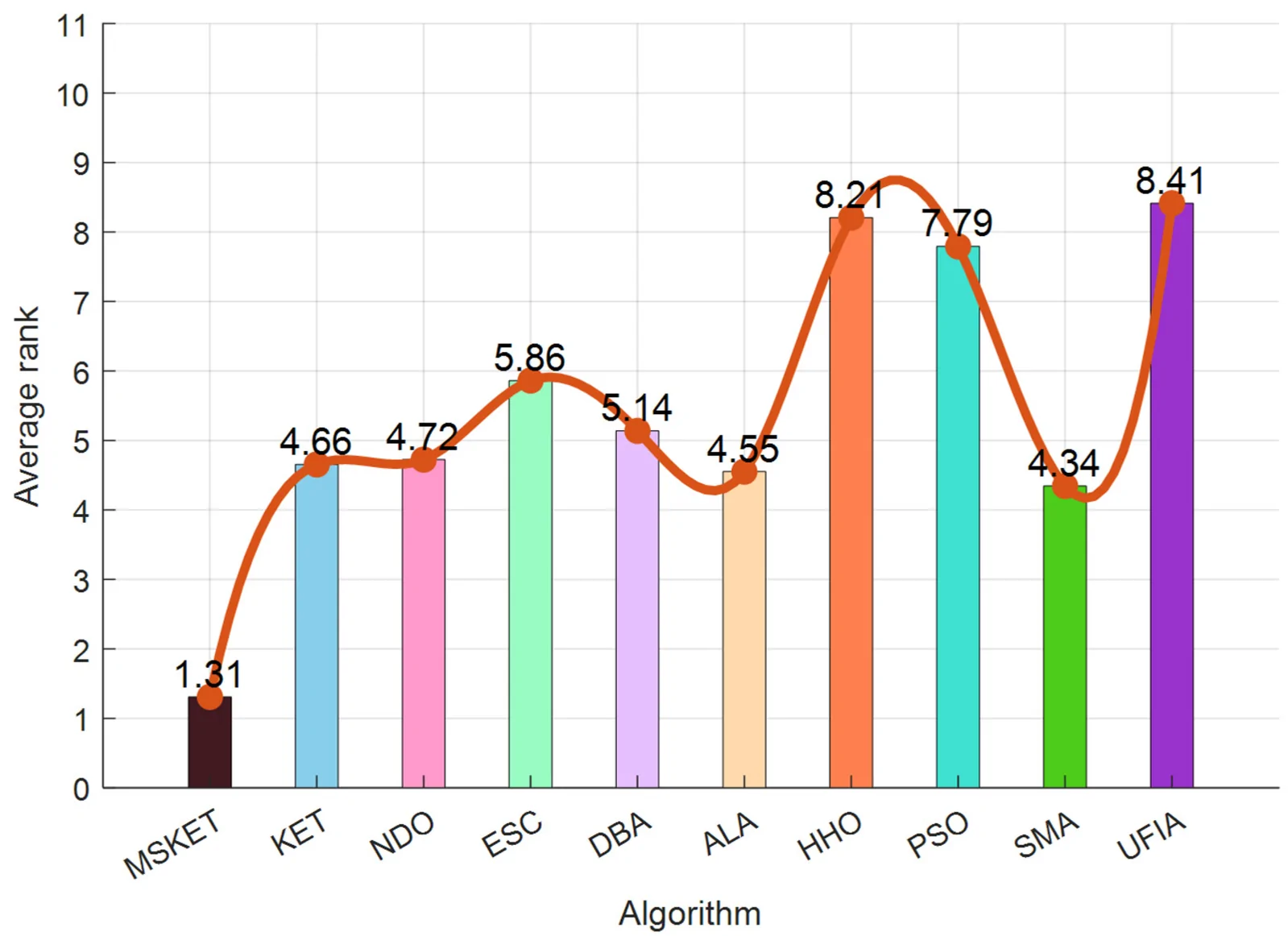
Average ranking chart of CEC2017 test functions.

Figure 8 shows the average ranking results of all algorithms on the CEC2017 test functions. A smaller average rank indicates better overall performance. MSKET obtains the lowest average rank of 1.31, ranking first among all algorithms. The average ranks of SMA, ALA, KET, and NDO are 4.34, 4.55, 4.66, and 4.72, respectively, showing relatively close performance among these four algorithms but a clear gap from MSKET.

DBA and ESC obtain average ranks of 5.14 and 5.86, respectively. PSO, HHO, and UFIA show higher average ranks of 7.79, 8.21, and 8.41, respectively. Overall, the average ranking results are consistent with the statistical comparisons in Table 6 and further support the leading performance of MSKET on the CEC2017 benchmark suite.

#### 3.2.5. Radar Chart Analysis of CEC2017

**Figure 9:**
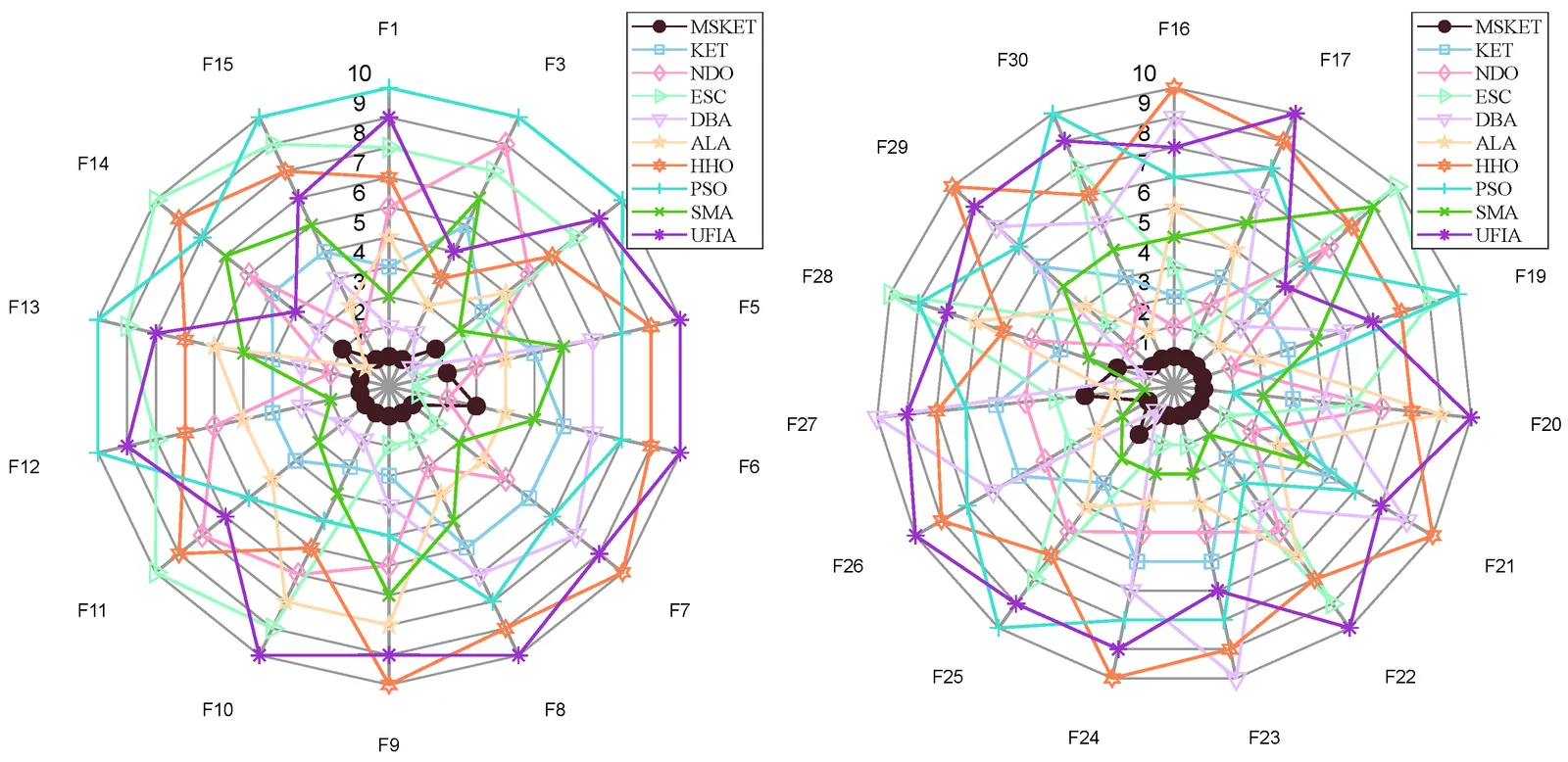
Radar charts of ranking results on CEC2017 test functions.

Figure 9 shows the radar charts of the ranking results on the CEC2017 test functions. The left chart corresponds to F1 and F3–F15, and the right chart corresponds to F16–F30. In the radar charts, a smaller rank and a position closer to the centre indicate better performance.

Overall, the ranking curve of MSKET is closer to the centre on most functions, especially on hybrid and composition functions. This indicates that MSKET maintains relatively stable rankings across different function types. In contrast, HHO, PSO, and UFIA are farther from the centre on several functions, showing lower rankings and larger fluctuations.

The radar charts are consistent with the Wilcoxon rank-sum test and average ranking results. Taken together, the CEC2017 results show that MSKET achieves strong optimization accuracy, stable convergence behaviour, and reliable performance across different high-dimensional test functions.

### 3.3. CEC2020 Test Functions

To further verify the performance of MSKET on another benchmark suite, all algorithms were tested on the 100-dimensional CEC2020 functions [18]. The following analysis covers statistical results, convergence curves, box plots, Wilcoxon rank-sum tests, average rankings, and radar charts.

#### 3.3.1. Analysis of Key Indicators for CEC2020

Table 7 reports the statistical results of all algorithms on the CEC2020 functions F1–F10, including the minimum value, standard deviation, mean value, and rank. MSKET ranks first on 8 of the 10 functions and ranks second and third on F5 and F6, respectively.

In terms of mean value, MSKET obtains the lowest values on F1–F4 and F7–F10. On F5 and F6, although MSKET does not achieve the best results, it remains within the top three. Compared with the original KET, MSKET obtains better rankings on all CEC2020 functions.

For the standard deviation, MSKET shows relatively small fluctuations on most functions, indicating stable results over 30 independent runs. Overall, the results in Table 7 show that MSKET maintains high ranking positions and stable performance on the CEC2020 benchmark suite.

#### 3.3.2. Convergence Analysis of CEC2020

**Figure 10:**
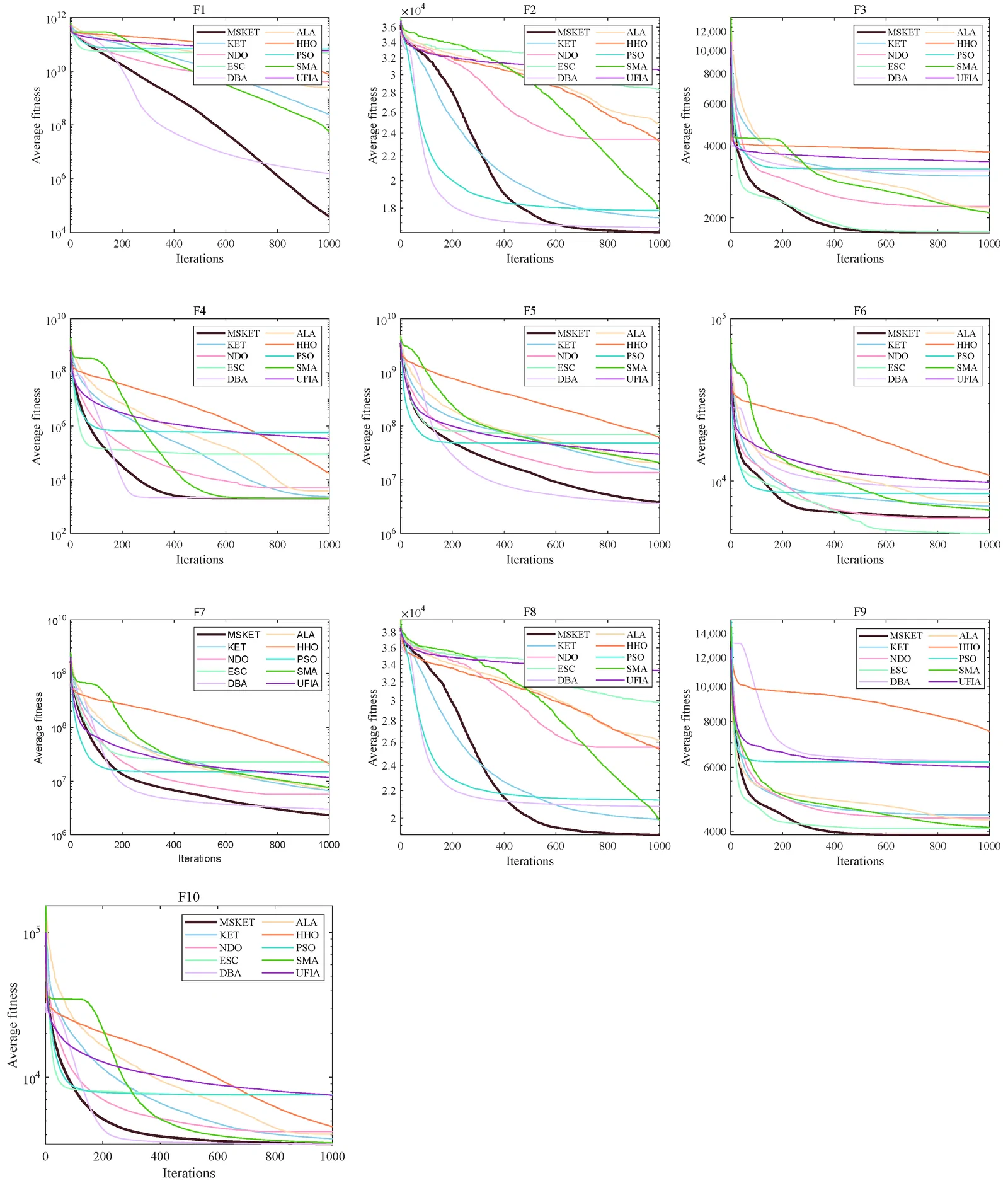
Mean convergence curves for CEC2020 functions F1–F10.

Figure 10 presents the mean convergence curves for CEC2020 functions F1–F10. Overall, MSKET decreases rapidly on most functions and reaches relatively low fitness values in the later iterations.

On F1, F2, and F3, MSKET shows clear downward trends. On F1, its curve decreases continuously throughout the iterations. On F2 and F3, MSKET continues to improve in the middle and late stages and obtains lower final fitness values than most comparison algorithms. Similar trends are observed on F4, F8, F9, and F10, where MSKET converges rapidly and remains at low fitness levels in the later stage.

On F5 and F7, MSKET is close to DBA during some iterations but still obtains competitive final results. On F6, MSKET does not achieve the best final convergence result, although its curve remains smooth and better than those of most comparison algorithms. Compared with the original KET, MSKET reaches lower final fitness values on several CEC2020 functions, showing improved convergence behaviour.

#### 3.3.3. Stability Analysis of CEC2020

**Figure 11:**
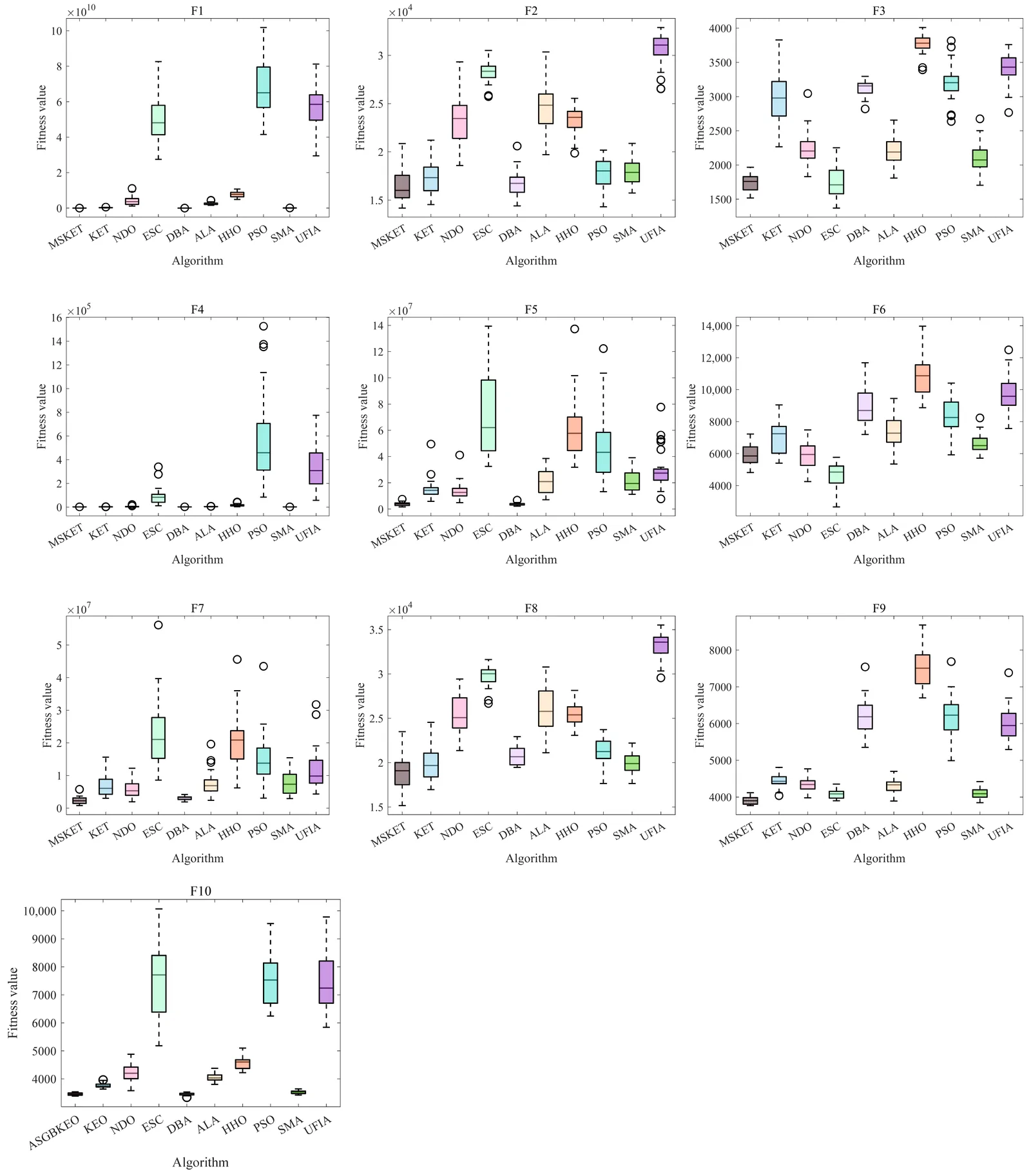
Box plots of CEC2020 test functions F1–F10. Circles in the box plots indicate outliers.

Figure 11 shows the box plots of all algorithms on the CEC2020 functions F1–F10 over 30 independent runs. Overall, MSKET presents low box positions and compact distributions on most functions.

MSKET performs well on F1, F3–F5, and F7–F10, where its median values are generally lower than those of most comparison algorithms. On F2, MSKET also remains at a relatively low box position, with only small differences from several competitive algorithms. On F6, although MSKET does not obtain the lowest box position, its distribution is still compact and shows no large fluctuation.

Several comparison algorithms show wider box spans or more outliers on some functions. In contrast, MSKET maintains compact result distributions on most CEC2020 functions, which is consistent with the statistical results in Table 7.

#### 3.3.4. Statistical Analysis of CEC2020

Table 8 reports the Wilcoxon rank-sum test results between MSKET and each comparison algorithm on the CEC2020 functions. Overall, MSKET obtains more “+” cases than “−” cases in all pairwise comparisons.

MSKET achieves 10/0/0 against ALA, HHO, PSO, and UFIA, showing significant advantages on all 10 functions. Compared with the original KET, MSKET obtains 9/0/1, with no significantly inferior case. The same result is observed in the comparisons with NDO and SMA.

For DBA, MSKET obtains 7/0/3, indicating significant advantages on most functions and no inferior case. Against ESC, the result is 8/1/1; although one “−” case appears, the number of “+” cases remains larger. In the final ranking, MSKET ranks first among all algorithms, which is consistent with the Wilcoxon test results.

**Figure 12:**
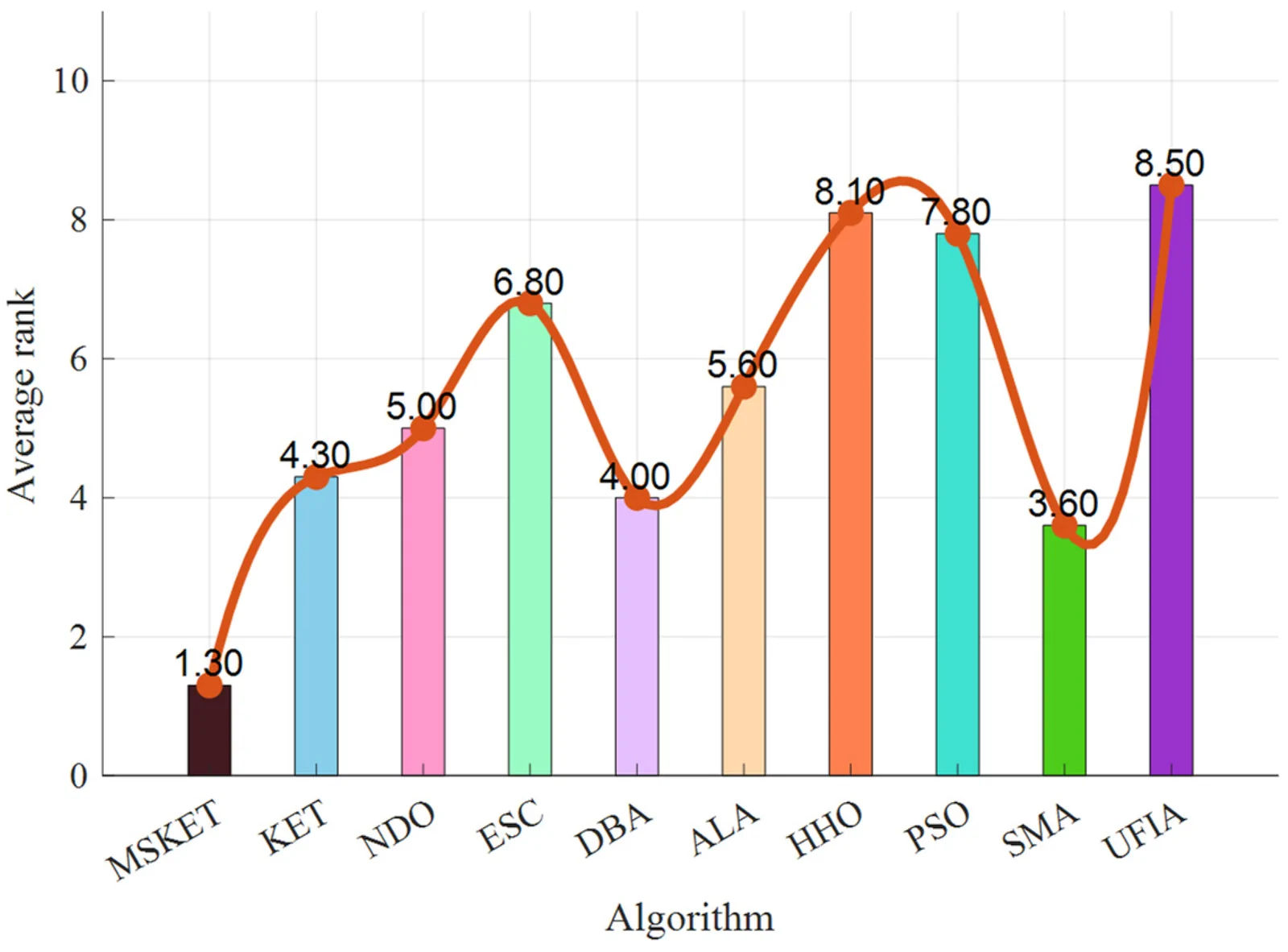
Average ranking chart of CEC2020 test functions.

Figure 12 shows the average ranking results of all algorithms on the CEC2020 functions. A smaller average rank indicates better overall performance. MSKET obtains the lowest average rank of 1.30, ranking first among all algorithms.

Compared with the original KET, the average rank decreases from 4.30 to 1.30, showing that MSKET achieves a better overall ranking on the CEC2020 benchmark suite. SMA and DBA obtain average ranks of 3.60 and 4.00, respectively, followed by KET, NDO, and ALA with average ranks of 4.30, 5.00, and 5.60. ESC, PSO, HHO, and UFIA have relatively higher average ranks.

Overall, the average ranking results are consistent with the statistical comparisons in Table 8 and support the leading position of MSKET on the CEC2020 functions.

#### 3.3.5. Radar Chart Analysis of CEC2020

**Figure 13:**
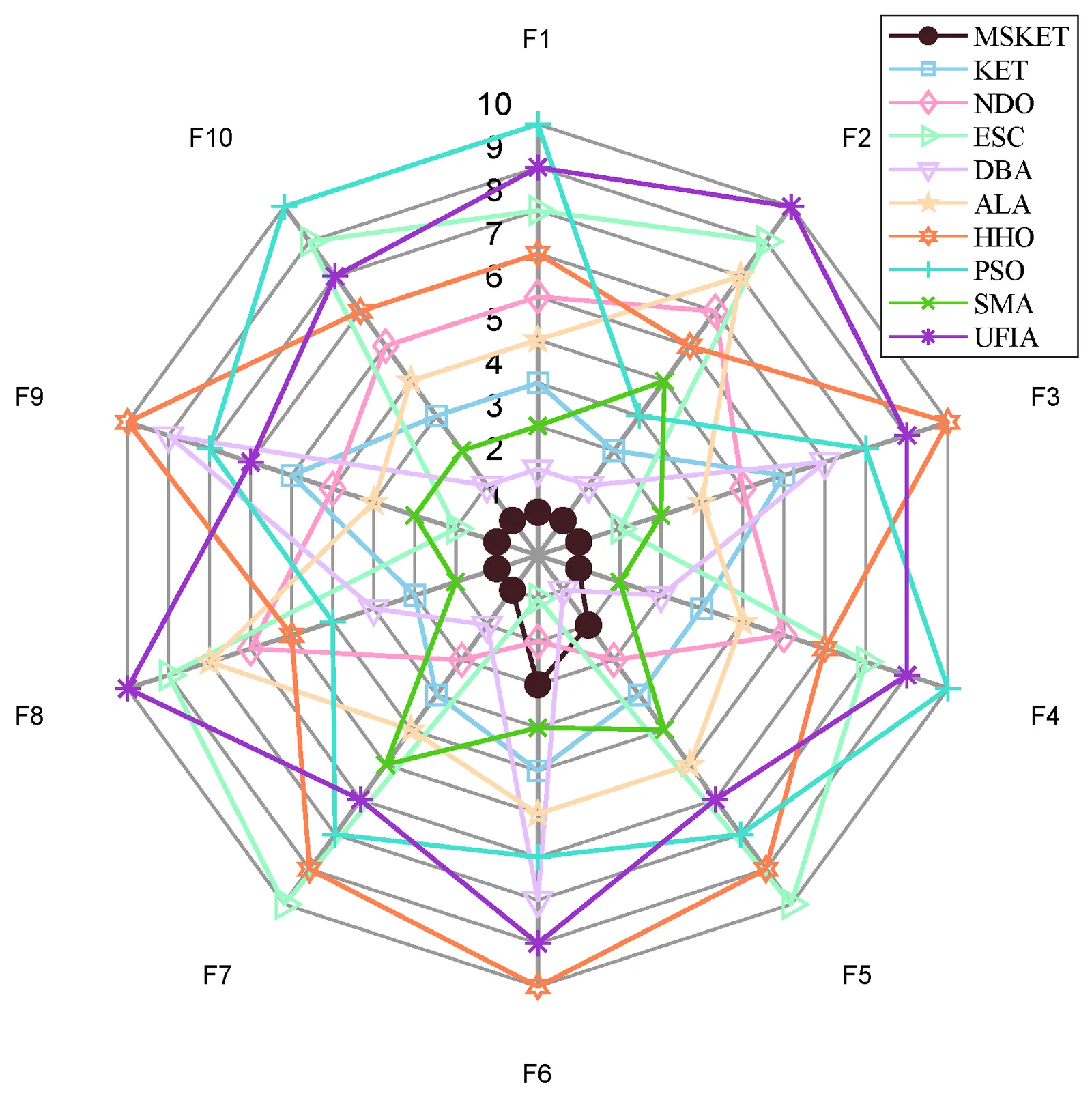
Radar chart of ranking results on CEC2020 test functions.

Figure 13 shows the radar chart of the ranking results on the CEC2020 functions F1–F10. In the radar chart, a smaller rank and a position closer to the centre indicate better performance on the corresponding function.

Overall, the ranking curve of MSKET is closer to the centre on most functions. MSKET stays near the centre on F1–F4 and F7–F10, corresponding to its leading ranks on these functions. On F5 and F6, although MSKET does not obtain the best rank, it still remains competitive.

By contrast, HHO, PSO, UFIA, and ESC are farther from the centre on several functions, reflecting lower ranks and larger ranking variations. The radar chart is consistent with the Wilcoxon test and average ranking results, showing that MSKET maintains stable ranking performance on the CEC2020 benchmark suite.

### 3.4. Ablation Study of MSKET

To assess the contribution of each component, seven variants were derived from the original KET. KET-AS, KET-CG, KET-DE, and KET-BD separately incorporate adaptive stage switching, candidate-group-based safe-area guidance, greedy DE/rand-to-best/1 refinement for low-ranked individuals, and beta-distribution opposition-based guidance. KET-AS-CG and KET-AS-CG-DE were included to examine cumulative effects, while MSKET contains all four components.

All variants were evaluated on the 100-dimensional CEC2017 and CEC2020 benchmark suites using the same settings as those in Section 3.2 and Section 3.3. The population size was 30, the maximum number of iterations was 1000, and each experiment was repeated 30 times. CEC2017 contained 29 functions after excluding F2, whereas CEC2020 contained 10 functions. Rankings were determined by the mean fitness value, with the standard deviation used to resolve ties. Table 9 summarizes the overall results.

The single-component variants generally improved KET, although their effects varied across the two suites. KET-BD achieved the best average rank among the single-component variants on CEC2017, whereas KET-DE performed best on CEC2020. KET-AS produced a smaller independent gain, particularly on CEC2020, indicating that the effectiveness of each component depends on the function landscape.

A clearer trend was observed for the cumulative variants. On CEC2017, the average rank decreased from 7.45 for KET to 4.03 for KET-AS-CG, 2.52 for KET-AS-CG-DE, and 1.07 for MSKET. The corresponding values on CEC2020 were 6.90, 4.70, 3.30, and 1.00. MSKET ranked first on 27 of the 29 CEC2017 functions and on all 10 CEC2020 functions. It ranked second on CEC2017 F10 and F22.

**Figure 14:**
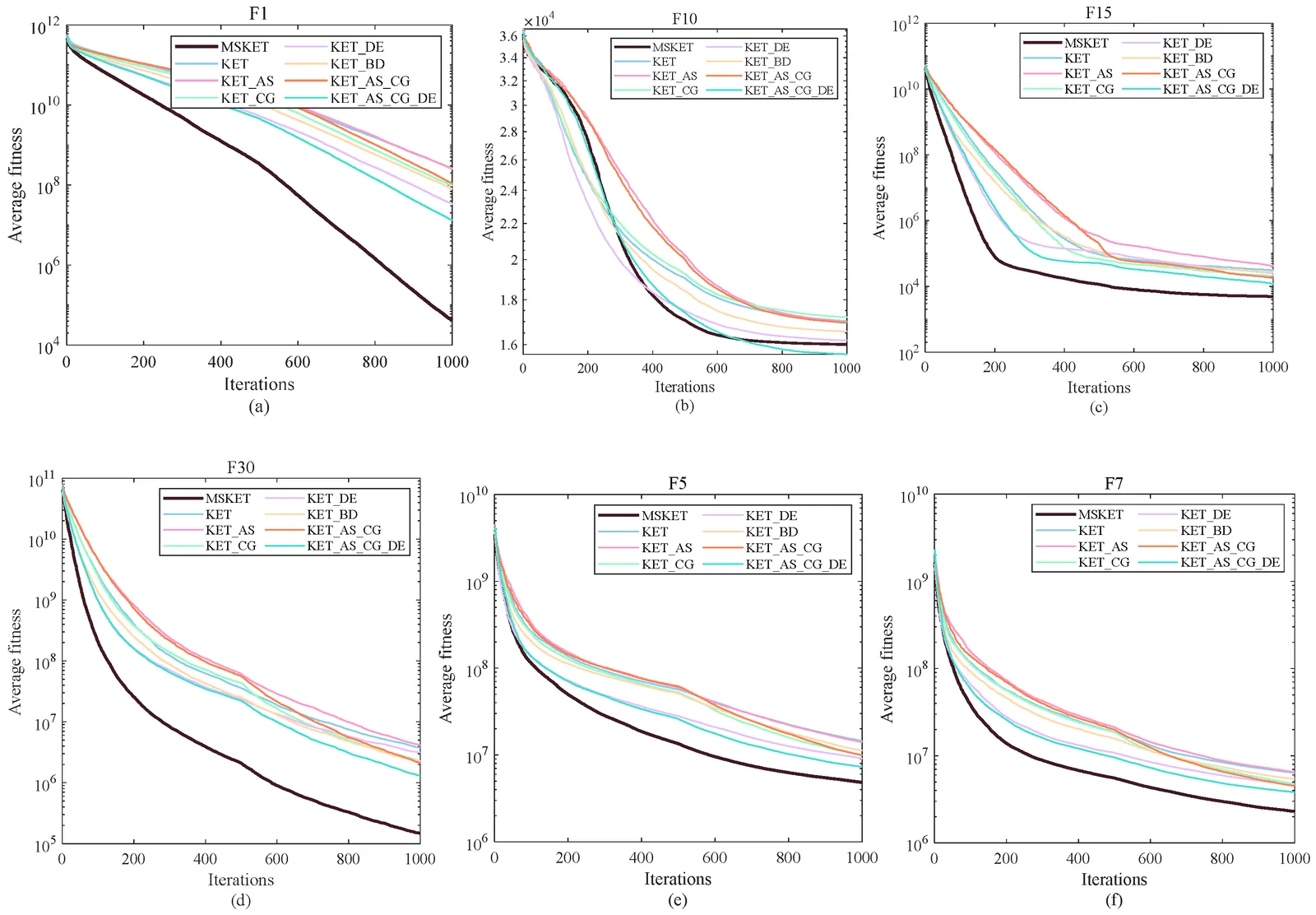
Mean convergence curves of the ablation variants on representative benchmark functions: (**a**) CEC2017 F1; (**b**) CEC2017 F10; (**c**) CEC2017 F15; (**d**) CEC2017 F30; (**e**) CEC2020 F5; and (**f**) CEC2020 F7.

The Friedman test [23,24] identified significant differences among the eight variants. The test statistics were $\chi_{F}^{2}(7)=131.4368$ for CEC2017 and $\chi_{F}^{2}(7)=37.2000$ for CEC2020, with corresponding *p*-values of 3.15 × 10^−25^ and 4.30 × 10^−6^, respectively. Figure 14 further shows that the cumulative variants generally achieved faster convergence or lower final fitness values than the original KET. These results indicate that the performance gain of MSKET arises from the complementary effects of the four components rather than from a single dominant strategy.

### 3.5. Population Diversity Analysis

To examine the population dynamics of MSKET, normalized population diversity was monitored throughout the optimization process. CEC2017 F1, F10, F15, and F30 were selected as representative unimodal, multimodal, hybrid, and composition functions, respectively. KET and MSKET were independently run 30 times under identical experimental settings.

At iteration *t*, diversity was quantified by the normalized mean pairwise distance defined in Equation (15):

(15) $$\mathrm{Div}(t)=\frac{2}{N(N-1)}\sum_{i=1}^{N-1}\sum_{j=i+1}^{N}\frac{{\left\|\left(x_{i}^{t}-x_{j}^{t}\right)⊘\left(ub-lb\right)\right\|}_{2}}{\sqrt{D}}$$

where *N* is the population size, *D* is the problem dimension, $x_{i}^{t}$ denotes the position of the *i*th individual at iteration *t*, and *ub* and *lb* are the upper and lower search bounds, respectively. The operator ⊘ denotes component-wise division. A larger value represents a more dispersed population, whereas a smaller value indicates greater concentration.

**Figure 15:**
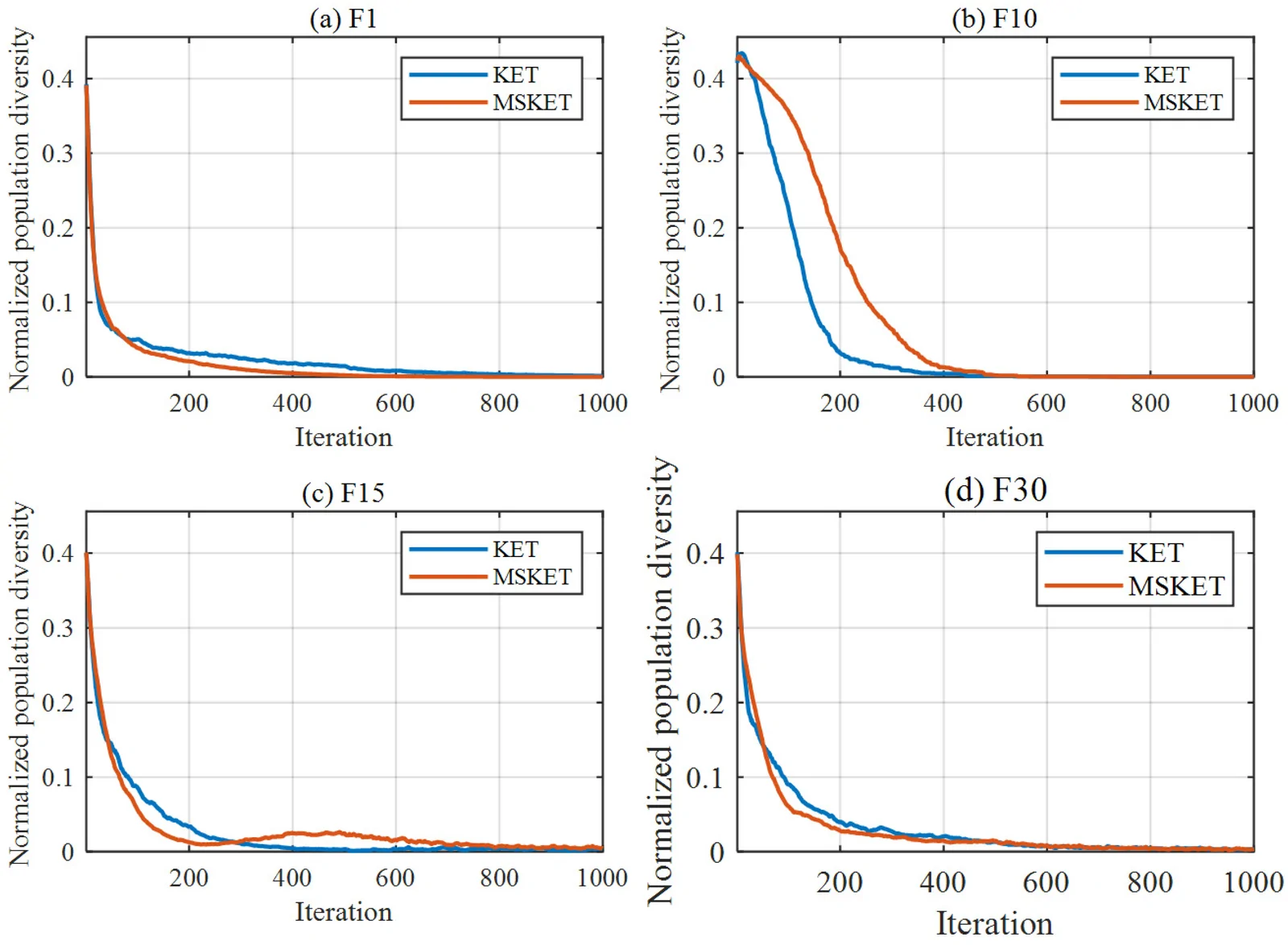
Mean normalized population diversity curves of KET and MSKET on representative CEC2017 functions: (**a**) F1; (**b**) F10; (**c**) F15; and (**d**) F30.

As shown in Figure 15, the diversity of KET declines rapidly on F1, F15, and F30, and subsequently remains at a relatively low level. By comparison, MSKET preserves a wider population spread during the early and middle stages before gradually contracting in the later iterations. This evolution is consistent with broader exploration at the beginning of the search and stronger exploitation toward the end.

A different trend is observed on F10. After the initial decline, the diversity of MSKET rises temporarily during the middle stage before decreasing again. The temporary recovery suggests renewed population dispersion in the multimodal landscape, while the subsequent decline reflects increasing concentration around promising regions.

The results show that MSKET does not simply maintain high diversity throughout the search. Instead, its population spread changes across iterations, with greater dispersion in the early and middle stages and stronger concentration near the end. Combined with the convergence results, this behaviour supports the improved regulation of exploration and exploitation in MSKET.

### 3.6. Computational Cost Analysis

The computational cost of KET and MSKET was assessed using objective-function evaluations (FEs), average CPU time, and computational complexity. Here, *N*, *T*, and *D* denote the population size, maximum number of iterations, and problem dimension, respectively. Consistent with the benchmark experiments, *N* = 30 and *T* = 1000.

KET evaluates the initial population once and performs *N* objective-function evaluations per iteration. For MSKET, each iteration includes *N* evaluations for the main position update, $N_{DE}$ evaluations for DE refinement, 2*N* evaluations in the implemented BD-OBL procedure, and *N* evaluations for fitness synchronization. The total FEs of the two algorithms are given by Equation (16):

(16) $$\begin{array}{l} {\mathrm{FEs}}_{\mathrm{KET}}=N+TN\\ {\mathrm{FEs}}_{\mathrm{MSKET}}=N+T\left(N+N_{\mathrm{DE}}+2N+N\right) \end{array}$$

The DE procedure refines individuals ranked from $\left\lceil 0.7N\right\rceil$ to *N*; therefore, $N_{DE}=N-\left\lceil 0.7N\right\rceil +1=10$ when *N* = 30. Substituting the experimental settings into Equation (16) gives 30,030 FEs for KET and 130,030 FEs for MSKET.

As shown in Table 10, MSKET requires approximately 4.33 times as many FEs as KET. Its average CPU time increases from 0.8511 s to 4.9531 s because of DE refinement, BD-OBL, and fitness synchronization. The comparisons in Section 3.2 and Section 3.3 were therefore conducted under an equal-iteration budget rather than an equal-FE budget.

Excluding the problem-dependent cost of objective-function evaluation, the dominant computational complexities are expressed in Equation (17):

(17) $$\begin{array}{l} C_{\mathrm{KET}}=O\left(TND\right)\\ C_{\mathrm{MSKET}}=O\left(TN^{2}D\right) \end{array}$$

The quadratic term in MSKET mainly arises from the pairwise population-distance calculation in BD-OBL. Thus, the improved search performance is accompanied by a measurable increase in computational cost.

### 3.7. Statistical Comparison

Overall differences among the algorithms were evaluated using the Friedman test. Rankings were based on the mean fitness value for each function, with lower ranks indicating better performance. The Iman–Davenport statistic was then calculated, followed by Nemenyi post-hoc comparisons at a significance level of 0.05 [23,24].

For CEC2017, the Friedman test yielded $\chi_{F}^{2}(9)=134.2665$ with a *p*-value of 1.57 × 10^−24^. The Iman–Davenport statistic was $F_{F}=29.6643$, with a *p*-value of 6.04 × 10^−35^. Both results reject the null hypothesis that all algorithms perform equivalently. MSKET achieved the lowest average rank of 1.3103. The Nemenyi critical difference was 2.5154, and the rank difference between MSKET and each of the other nine algorithms exceeded this threshold. Thus, MSKET significantly outperformed all comparison algorithms on CEC2017.

For CEC2020, the Friedman statistic was $\chi_{F}^{2}(9)=52.2982$, with a *p*-value of 3.97 × 10^−8^. The corresponding Iman–Davenport statistic was $F_{F}=12.4844$, with a *p*-value of 3.46 × 10^−12^. These results also indicate significant overall differences among the algorithms. MSKET again obtained the lowest average rank of 1.3000. The Nemenyi critical difference was 4.2836. The rank differences between MSKET and ALA, ESC, PSO, HHO, and UFIA exceeded this value, whereas those involving KET, NDO, DBA, and SMA did not reach the significance threshold.

Overall, MSKET achieved the best average rank on both benchmark suites. The Friedman and Iman–Davenport tests confirmed significant overall differences, while the Nemenyi analysis showed broader statistical superiority on CEC2017 than on CEC2020. These results provide additional statistical support for the performance improvements observed in the Wilcoxon tests and ranking analysis.

## 4. Engineering Design and NIR Prediction Applications

For the piston–lever and three-bar truss design problems, all algorithms used a population size of 30 and a maximum of 500 iterations, with each experiment repeated independently 30 times.

### 4.1. Piston–Lever Design Problem

**Figure 16:**
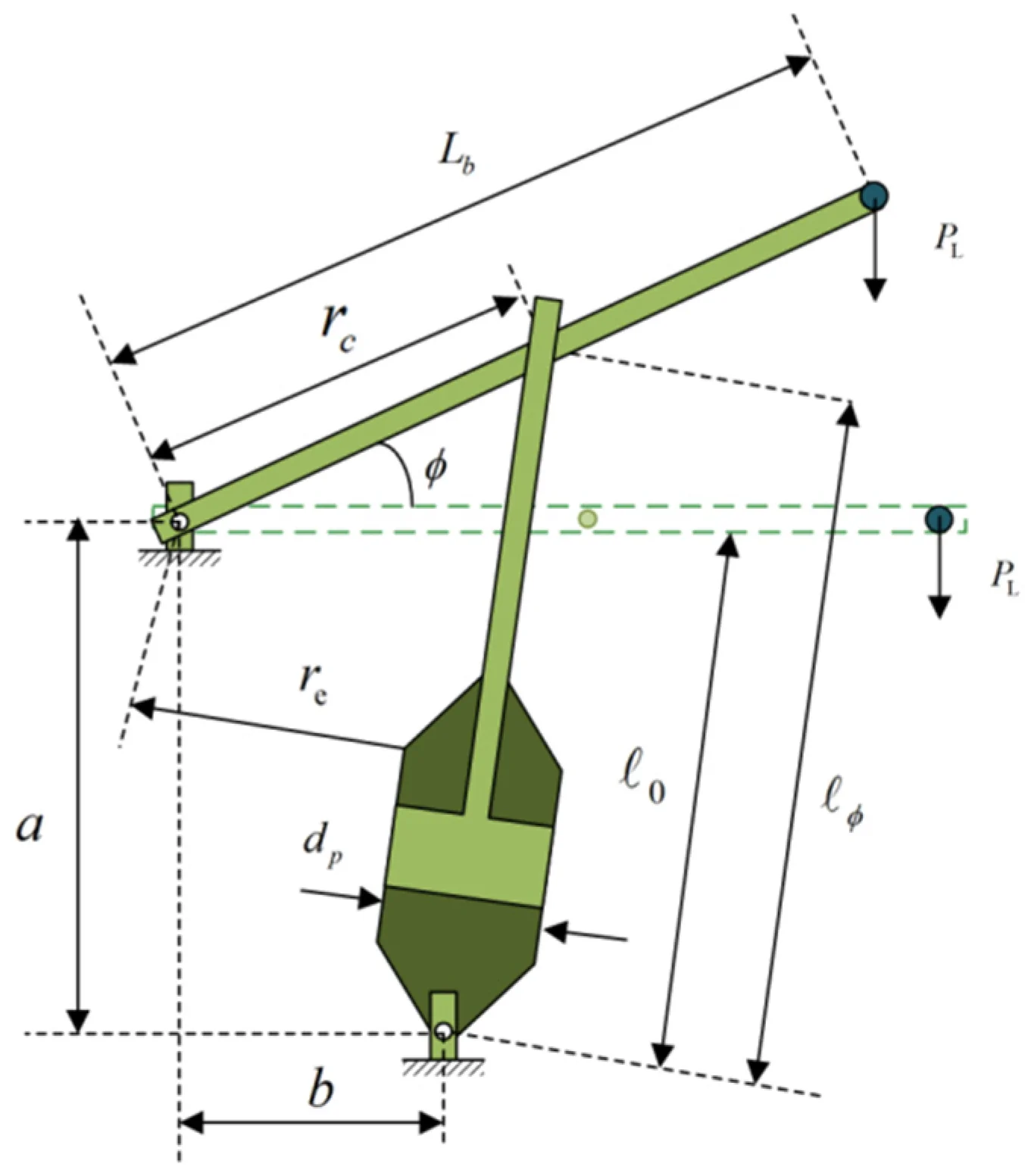
Schematic of the piston–lever mechanism.

The piston–lever design problem was selected to assess the performance of MSKET in constrained mechanical design. As illustrated in Figure 16, the geometric parameters of the piston–lever mechanism are optimized to minimize the oil volume displaced when the lever rotates from 0° to 45°. The design must satisfy the force-equilibrium, bending-moment, piston-stroke, and geometric constraints. The original problem contains four continuous design variables and four nonlinear inequality constraints [25]. The design vector is defined in Equation (18):

(18) $$z={\left[a,\;b,\;d_{p},\;r_{c}\right]}^{T}$$

where *a* and *b* are the vertical and horizontal installation offsets, respectively, $d_{p}$ is the piston diameter, and $r_{c}$ denotes the piston connection position along the lever. The remaining geometric quantities in Figure 16 include the lever length $L_{b}$, rotation angle ϕ, effective moment arm $r_{e}$, and piston lengths $\ell_{0}$ and $\ell_{\phi }$. The objective function, defined as the displaced oil volume, is expressed in Equation (19):

(19) $$\min\;V(z)=\frac{\pi d_{p}^{2}}{4}\left(\ell_{\phi }-\ell_{0}\right)$$

where $\ell_{0}$ and $\ell_{\phi }$ are the piston lengths at the initial and rotated positions, respectively. They are determined using Equation (20):

(20) $$\ell_{0}=\sqrt{{\left(r_{c}-b\right)}^{2}+a^{2}},\;\;\ell_{\phi }=\sqrt{{\left(r_{c}\sin\phi +a\right)}^{2}+{\left(b-r_{c}\cos\phi \right)}^{2}}$$

The effective moment arm and hydraulic thrust are expressed in Equation (21):

(21) $$r_{e}=\frac{\left|-r_{c}\left(r_{c}\sin\phi +a\right)+a\left(b-r_{c}\cos\phi \right)\right|}{\sqrt{{\left(r_{c}-b\right)}^{2}+a^{2}}},\;\;F_{h}=\frac{\pi p_{h}d_{p}^{2}}{4}$$

The mechanical and geometric requirements are summarized in Equation (22):

(22) $$\begin{array}{l} c_{1}(z)=P_{L}L_{b}\cos\phi -r_{e}F_{h}\leq 0,\\ c_{2}(z)=P_{L}\left(L_{b}-r_{c}\right)-M_{\lim}\leq 0,\\ c_{3}(z)=1.2\left(\ell_{\phi }-\ell_{0}\right)-\ell_{0}\leq 0,\\ c_{4}(z)=\frac{d_{p}}{2}-b\leq 0. \end{array}$$

In Equation (22), $c_{1}$, $c_{2}$, $c_{3}$, and $c_{4}$ represent the force-equilibrium, allowable bending-moment, minimum piston-stroke, and geometric constraints, respectively. The fixed parameters are given in Equation (23):

(23) $$\begin{array}{l} \phi =45{}^{\circ},P_{L}=10,000\;\mathrm{lb},L_{b}=240\;\mathrm{in},\\ M_{\lim}=1.8\times 10^{6}\;\mathrm{lb}\cdot \mathrm{in},p_{h}=1500\;\mathrm{psi} \end{array}$$

The allowable ranges of the design variables are specified in Equation (24):

(24) $$0.05\leq a,b,r_{c}\leq 500,\;\;0.05\leq d_{p}\leq 120$$

Equations (18)–(24) define a constrained optimization model with four continuous variables and four nonlinear inequality constraints. The objective is to minimize the displaced oil volume subject to force-equilibrium, bending-moment, piston-stroke, and geometric requirements.

**Figure 17:**
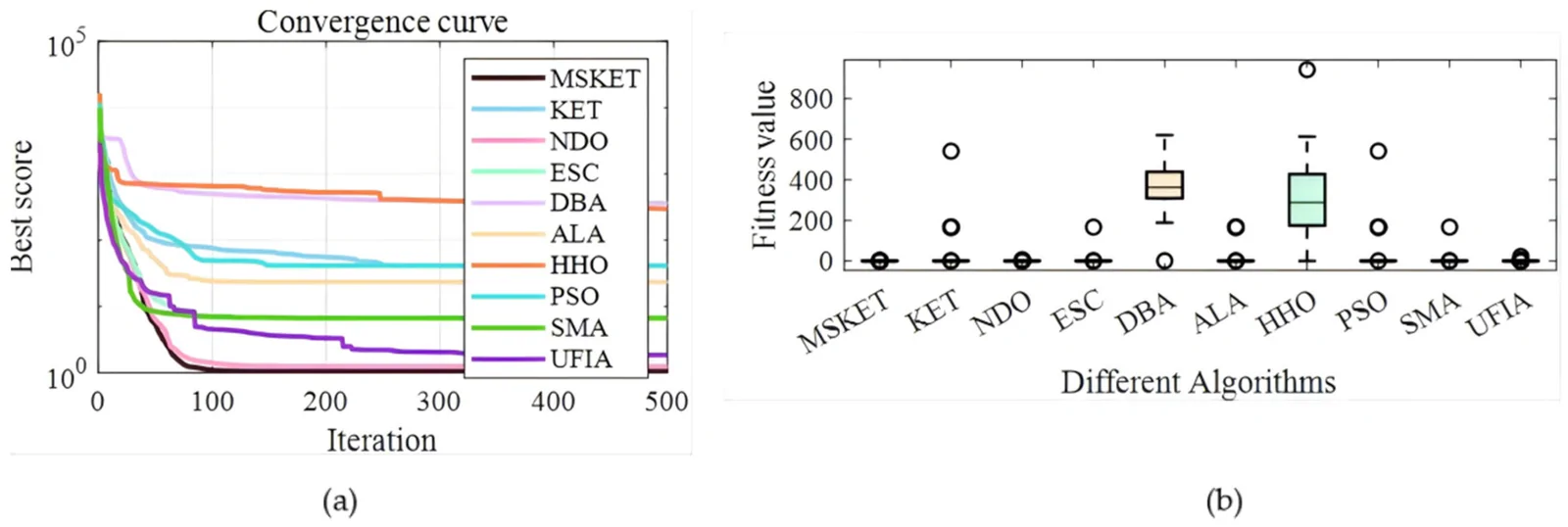
Optimization results for the piston–lever design problem: (**a**) mean convergence curves; (**b**) box plots of the final objective values. Circles in the box plots indicate outliers.

MSKET and the nine comparison algorithms were independently executed 30 times under the same population size, iteration budget, and constraint-handling procedure. Table 11 reports the best objective value, mean objective value, and standard deviation obtained by each algorithm over 30 independent runs. The average convergence curves and box plots of the final objective values are presented in Figure 17a and Figure 17b, respectively.

As reported in Table 11, the optimal design obtained by MSKET is given in Equation (25):

(25) $$z^{*}={\left[0.05,\;1.0081135174,\;2.0162270347,\;500\right]}^{T}$$

The corresponding minimum objective value was 1.057393888. The mean value was also 1.057393888 to the reported precision, and the standard deviation was only 7.81 × 10^−14^, placing MSKET first according to the mean objective value. Although several algorithms obtained comparable minimum values, their repeated-run performance was less stable.

Figure 17a shows that MSKET rapidly converged to the lowest objective region. The highly concentrated distribution in Figure 17b is consistent with its small standard deviation. In contrast, KET obtained a mean value of 41.27169275 and a standard deviation of 110.5924767. These results show that MSKET markedly improved the repeated-run stability of KET and achieved the best overall statistical performance among the tested algorithms.

### 4.2. Three-Bar Truss Design Problem

**Figure 18:**
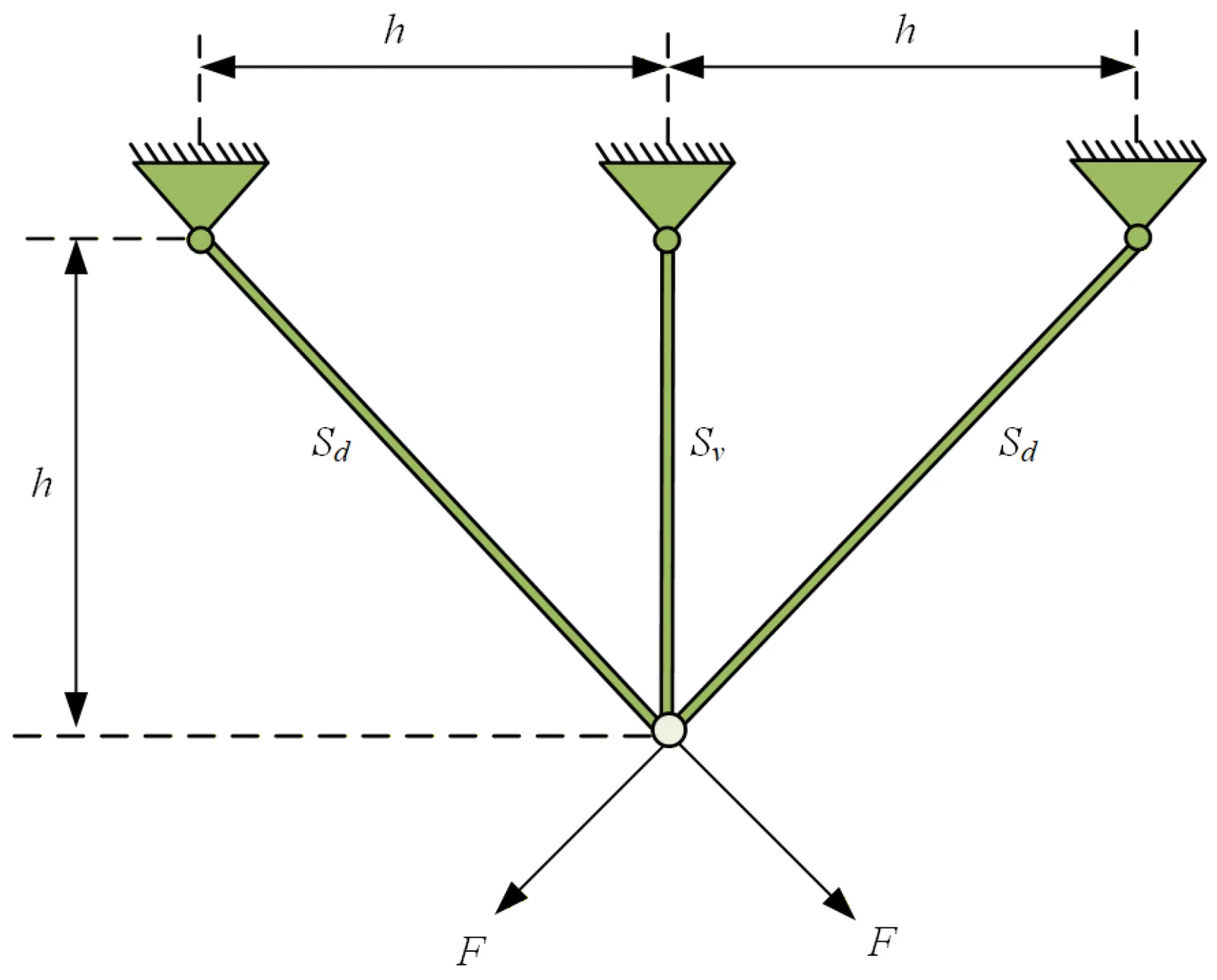
Schematic configuration and design variables of the three-bar truss.

The three-bar truss design problem was used to evaluate the performance of MSKET in constrained structural optimization. As shown in Figure 18, the structure consists of two symmetric diagonal members and one vertical member. The objective is to minimize the structural volume by determining the cross-sectional areas of the members while satisfying the allowable-stress constraints [25].

The design vector is defined in Equation (26):

(26) $$\eta ={\left[S_{d},\;S_{v}\right]}^{T}$$

where $S_{d}$ denotes the cross-sectional area shared by the two diagonal members, and $S_{v}$ is the cross-sectional area of the vertical member. In Figure 18, h denotes both the vertical height of the truss and the horizontal distance between adjacent upper joints, while *F* represents each external load applied at the lower joint.

The structural volume is expressed in Equation (27):

(27) $$\min\;J_{T}(\eta )=\left(2\sqrt{2}S_{d}+S_{v}\right)h$$

The allowable-stress constraints are formulated in Equation (28):

(28) $$\begin{array}{l} \psi_{1}(\eta )=\frac{F\left(\sqrt{2}S_{d}+S_{v}\right)}{\sqrt{2}S_{d}^{2}+2S_{d}S_{v}}-\sigma_{a}\leq 0,\\ \psi_{2}(\eta )=\frac{FS_{v}}{\sqrt{2}S_{d}^{2}+2S_{d}S_{v}}-\sigma_{a}\leq 0,\\ \psi_{3}(\eta )=\frac{F}{S_{d}+\sqrt{2}S_{v}}-\sigma_{a}\leq 0. \end{array}$$

In Equation (28), $\psi_{1}$, $\psi_{2}$, and $\psi_{3}$ restrict the stresses in the truss members to the allowable stress $\sigma_{a}$. The fixed parameters and design-variable bounds are specified in Equation (29):

(29) $$h=100\;\mathrm{cm},\;\;F=2\;\mathrm{kN},\;\;\sigma_{a}=2\;{\mathrm{kN}/\mathrm{cm}}^{2},\;\;0\leq S_{d},S_{v}\leq 1\;{\mathrm{cm}}^{2}$$

**Figure 19:**
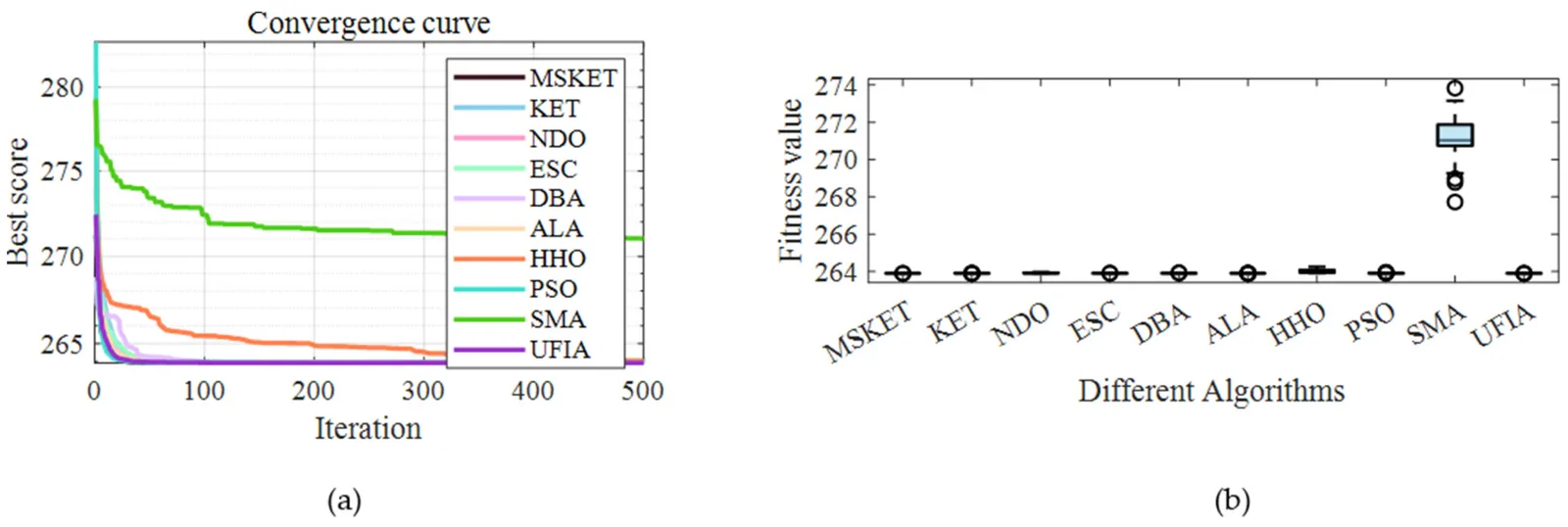
Optimization results for the three-bar truss design problem: (**a**) mean convergence curves; (**b**) box plots of the final objective values. Circles in the box plots indicate outliers.

Equations (26)–(29) define a constrained structural optimization model with two continuous variables and three nonlinear inequality constraints. The objective is to minimize the structural volume while maintaining the stresses in all members within the allowable limit. MSKET and the nine comparison algorithms were independently executed 30 times under the same experimental conditions. Table 12 reports the best objective value, mean objective value, and standard deviation obtained by each algorithm over 30 independent runs. The average convergence curves and box plots of the final objective values are presented in Figure 19a and Figure 19b, respectively.

As reported in Table 12, the optimal design obtained by MSKET is given in Equation (30):

(30) $$\eta^{*}={\left[0.7886751348,\;0.4082482899\right]}^{T}$$

The corresponding objective value was 263.895843376464. MSKET obtained the same average value as the reported precision, with a standard deviation of 1.78 × 10^−13^, and ranked first according to the average objective value. Although KET and ALA reached comparable best values, MSKET produced a more concentrated distribution over the 30 independent runs. Figure 19a shows that MSKET converged to the lowest objective region. The compact distribution in Figure 19b is consistent with its small standard deviation. These results indicate that MSKET achieved the best overall statistical performance and reliable repeated-run behaviour on the three-bar truss design problem.

### 4.3. Near-Infrared Prediction of Praeruptorin Content

To further verify the applicability of MSKET in practical regression prediction tasks, MSKET was combined with a BP neural network to construct the MSKET-BP prediction model and applied to near-infrared spectral modelling of white-flowered *Peucedanum praeruptorum*. Previous studies have shown that near-infrared spectroscopy combined with chemometric or regression models can be used for the rapid quantitative analysis of praeruptorins and related quality indicators in Peucedani Radix [26,27]. Therefore, in this study, near-infrared spectral variables were used as inputs, and the total content of praeruptorin A and praeruptorin B was used as the output to evaluate the modelling performance of MSKET-BP in predicting active component content in traditional Chinese medicinal materials.

#### 4.3.1. Dataset Description and Preprocessing

**Figure 20:**
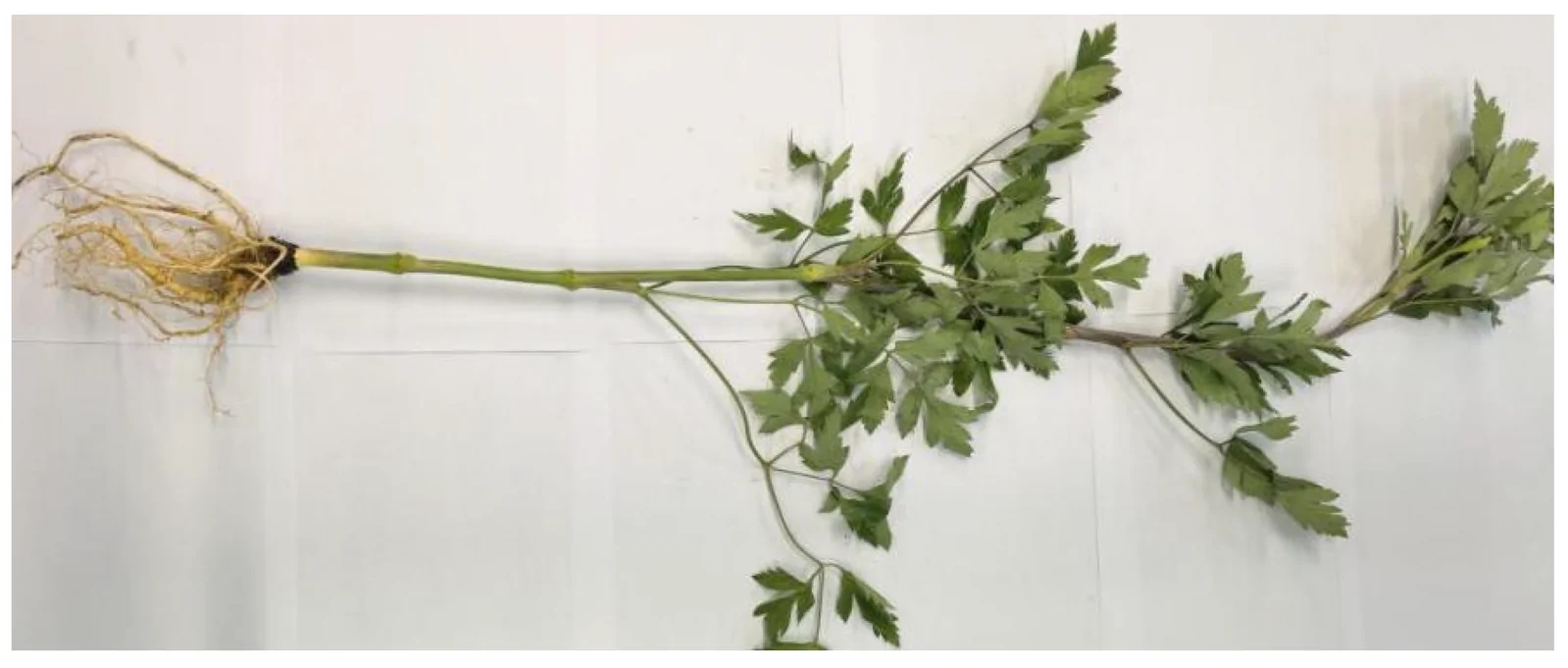
Sample of white-flowered *Peucedanum praeruptorum*.

Near-infrared (NIR) spectral data from white-flowered *Peucedanum praeruptorum* samples were used to evaluate the predictive performance of MSKET-BP. Spectroscopic techniques combined with chemometrics have been widely applied to the quality control and component analysis of traditional Chinese medicine [28]. The NIR spectral variables were used as the model inputs, whereas the total content of praeruptorin A and praeruptorin B was used as the response variable. A representative sample of white-flowered *P. praeruptorum* is shown in Figure 20.

**Figure 21:**
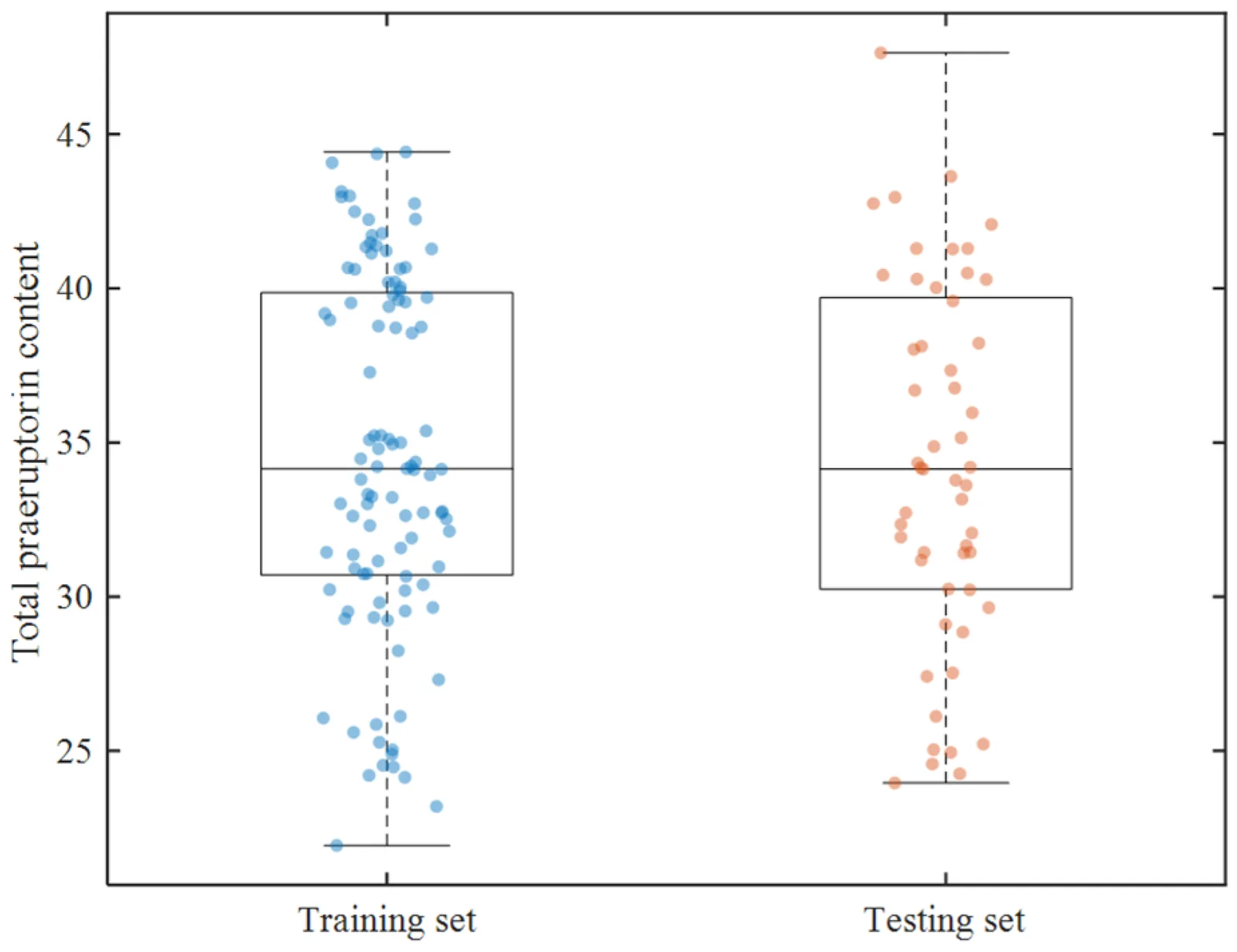
Distribution of the total content of praeruptorin A and praeruptorin B in the training and testing sets (blue: training set; orange: testing set).

The dataset contained 157 samples, including 104 training samples and 53 testing samples. Each sample comprised 119 NIR spectral variables and one response value. The first column contained the total content of praeruptorin A and praeruptorin B, whereas the remaining columns contained the spectral variables. Figure 21 presents the response-value distributions of the training and testing sets. The two subsets showed similar ranges and median values, indicating no marked distribution shift in the response variable between them.

**Figure 22:**
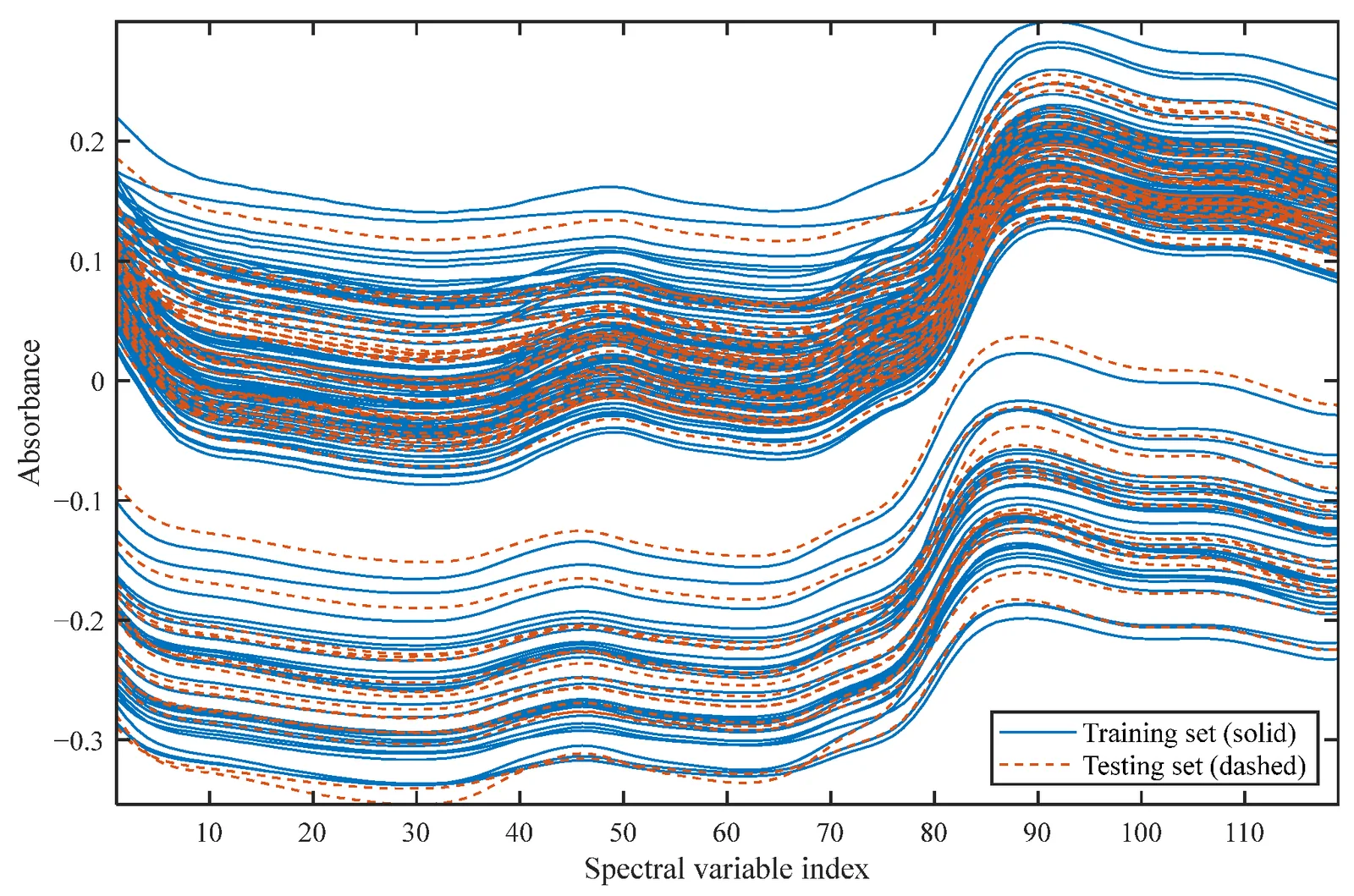
Near-infrared spectra of white-flowered *Peucedanum praeruptorum* samples (blue solid lines: training set; red dashed lines: testing set).

The NIR spectra of the samples are shown in Figure 22. The spectral curves exhibited similar overall profiles, together with local variations at different spectral-variable positions. These variations provided the spectral information required to model the relationship between the NIR variables and total praeruptorin content.

Before model construction, empty rows and columns were removed from the training and testing datasets. The spectral variables and response values were then normalized to the interval [0, 1] to reduce differences in numerical scale during network training. The normalized training set was used for model fitting, whereas the testing set was used to evaluate prediction performance on unseen samples.

#### 4.3.2. MSKET-BP Model Construction

A backpropagation neural network was used to model the nonlinear relationship between the NIR spectral variables and total praeruptorin content [29]. Metaheuristic algorithms have been widely used to optimize neural network weights and biases [30]. Neural network models combined with NIR spectroscopy have also been applied to the quality control of traditional Chinese medicine extracts [31]. Therefore, MSKET was adopted to optimize the initial weights and biases of the BP network.

The input layer contained 119 spectral variables, and the output layer contained one node corresponding to the total content of praeruptorin A and praeruptorin B. The network structure was set as 119–5–1, with five hidden neurons.

In MSKET-BP, each individual encodes a complete set of BP initial parameters, including the input-to-hidden weights, hidden-layer biases, hidden-to-output weights, and output-layer bias. The encoding form is given in Equation (31).

(31) $$x=\left[vec(w_{1}),b_{1},vec(w_{2}),b_{2}\right]$$

where $w_{1}$ and $w_{2}$ are the weight matrices of the input-to-hidden and hidden-to-output layers, respectively. $b_{1}$ and $b_{2}$ are the hidden-layer and output-layer biases. The corresponding optimization dimension is calculated by Equation (32).

(32) $$D=n_{in}n_{h}+n_{h}+n_{h}n_{out}+n_{out}$$

where $n_{in}$, $n_{h}$, and $n_{out}$ denote the numbers of input nodes, hidden neurons, and output nodes, respectively. In this study, $n_{in}=119$, $n_{h}=5$, and $n_{out}=1$. Thus, the dimension of the optimization problem is shown in Equation (33).

(33) D=119×5+5+5×1+1=606

During optimization, each MSKET individual was decoded into BP weights and biases. The decoded network was evaluated on the normalized training set, and the mean squared error was used as the fitness value, as shown in Equation (34).

(34) $$f(x)=\frac{1}{N_{tr}}\sum_{i=1}^{N_{tr}}{\left({\hat{y}}_{i}-y_{i}\right)}^{2}$$

where $N_{tr}$ is the number of training samples, $y_{i}$ is the normalized measured value of the *i*-th sample, and ${\hat{y}}_{i}$ is the corresponding network output. After MSKET obtained the best individual, it was used as the initial parameter set of the BP network for subsequent training.

The population size of MSKET was 20, the maximum number of iterations was 100, and the search range was [−1, 1]. The BP network was trained with a maximum of 1000 epochs, a target error of $1\times 10^{-6}$, and a learning rate of 0.01. After training, the testing predictions were transformed back to the original scale by inverse normalization.

#### 4.3.3. Evaluation Metrics

The testing performance of MSKET-BP was assessed using the coefficient of determination $R^{2}$, root mean square error (RMSE), and mean absolute percentage error (MAPE). $R^{2}$ measures the fitting degree between measured and predicted values, while RMSE and MAPE quantify prediction errors from absolute and relative perspectives. These indicators are defined in Equation (35).

(35) $$R^{2}=1-\frac{\sum_{i=1}^{N}e_{i}^{2}}{\sum_{i=1}^{N}{\left(y_{i}-\bar{y}\right)}^{2}},RMSE=\sqrt{\frac{1}{N}\sum_{i=1}^{N}e_{i}^{2}},MAPE=\frac{100\%}{N}\sum_{i=1}^{N}\left|\frac{e_{i}}{y_{i}}\right|,$$

Here, *N* denotes the number of testing samples; $y_{i}$ and ${\hat{y}}_{i}$ are the measured and predicted values for sample *i*, respectively; $\bar{y}$ is the mean of the measured values; and $e_{i}=y_{i}-{\hat{y}}_{i}$ is the prediction residual.

#### 4.3.4. Prediction Results and Analysis

The prediction results of different models on the testing set are shown in Table 13. The conventional BP model showed relatively weak prediction performance, with $R^{2}$, RMSE, and MAPE values of 0.61992, 3.5789, and 7.7638%, respectively. In contrast, all optimized BP models achieved better prediction results, indicating that optimizing the initial weights and biases of the BP neural network can effectively improve its regression prediction performance.

Among all models, MSKET-BP achieved the best prediction performance, with an $R^{2}$ of 0.86440, an RMSE of 2.1377, and a MAPE of 5.0083%. Compared with the conventional BP model, MSKET-BP increased $R^{2}$ by 0.24448 and reduced RMSE and MAPE by approximately 40.27% and 35.49%, respectively. Compared with the other optimized BP models, MSKET-BP also showed better overall performance. Among the comparative models, SMA-BP achieved a relatively high $R^{2}$ and a low RMSE, but its $R^{2}$ and RMSE values were 0.83048 and 2.3901, respectively, which were still inferior to those of MSKET-BP. In addition, the MAPE of ALA-BP was 5.2561%, which was higher than that of MSKET-BP. These results indicate that MSKET can further improve the prediction accuracy and generalization ability of the BP neural network by optimizing its initial parameters.

**Figure 23:**
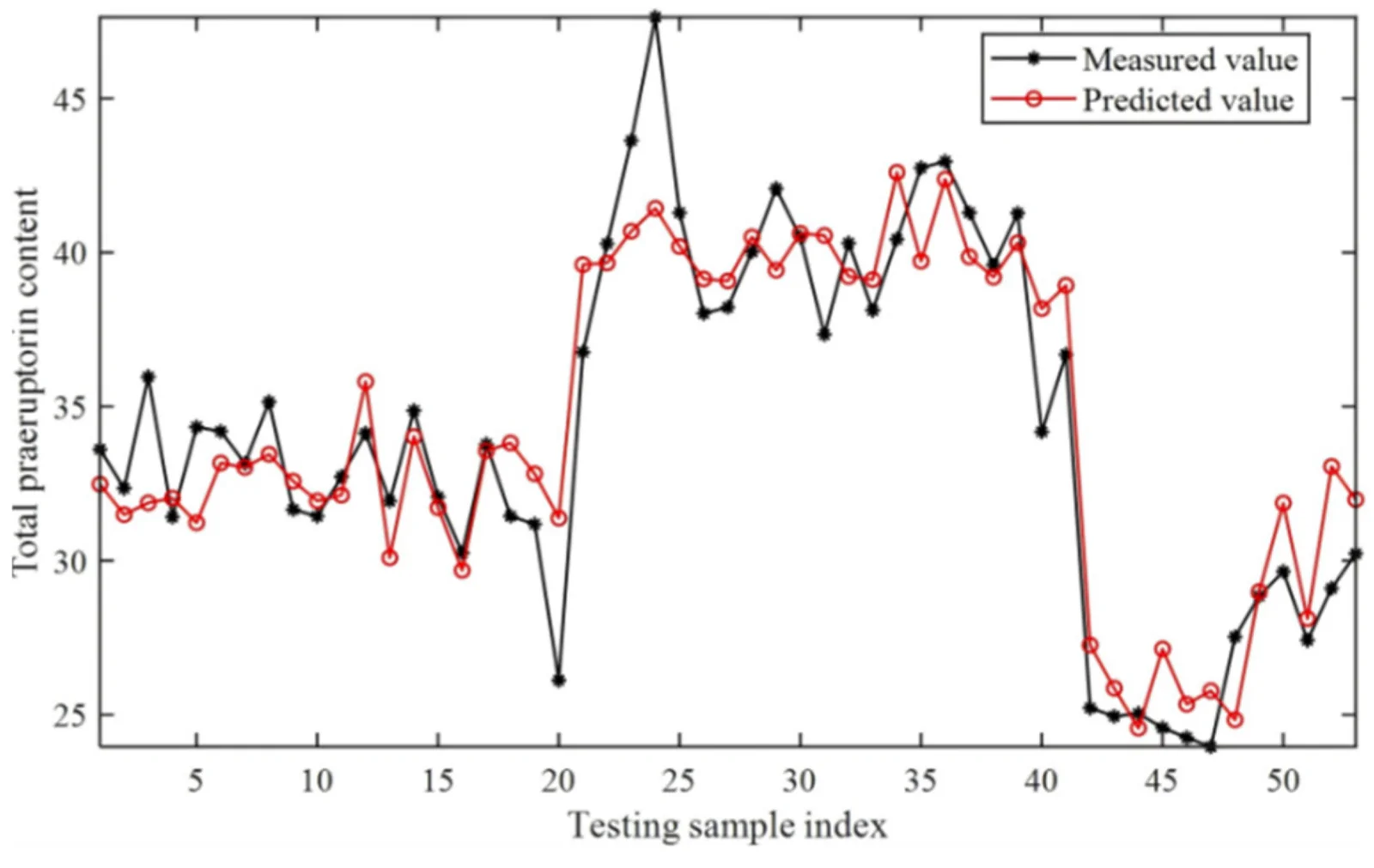
Comparison between measured and predicted values of MSKET-BP on the testing set.

The comparison between the measured and predicted values on the testing set is shown in Figure 23. The predicted curve of MSKET-BP generally follows the variation trend of the measured values, and the predicted values are close to the measured values for most testing samples.

**Figure 24:**
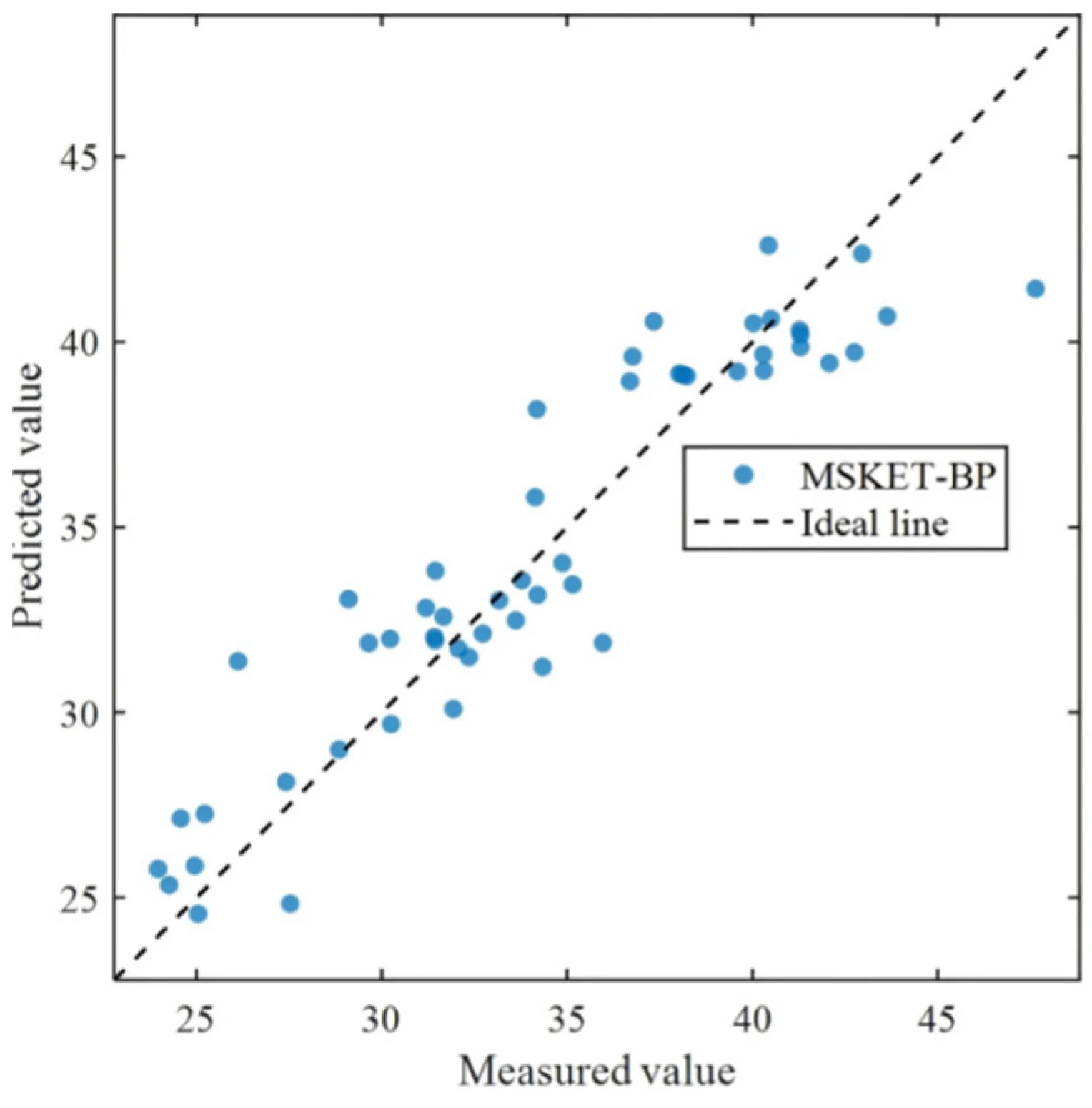
Scatter plot of measured and predicted values of MSKET-BP on the testing set.

Figure 24 presents the scatter plot of the measured and predicted values. Most sample points are distributed near the ideal line, indicating good prediction consistency.

**Figure 25:**
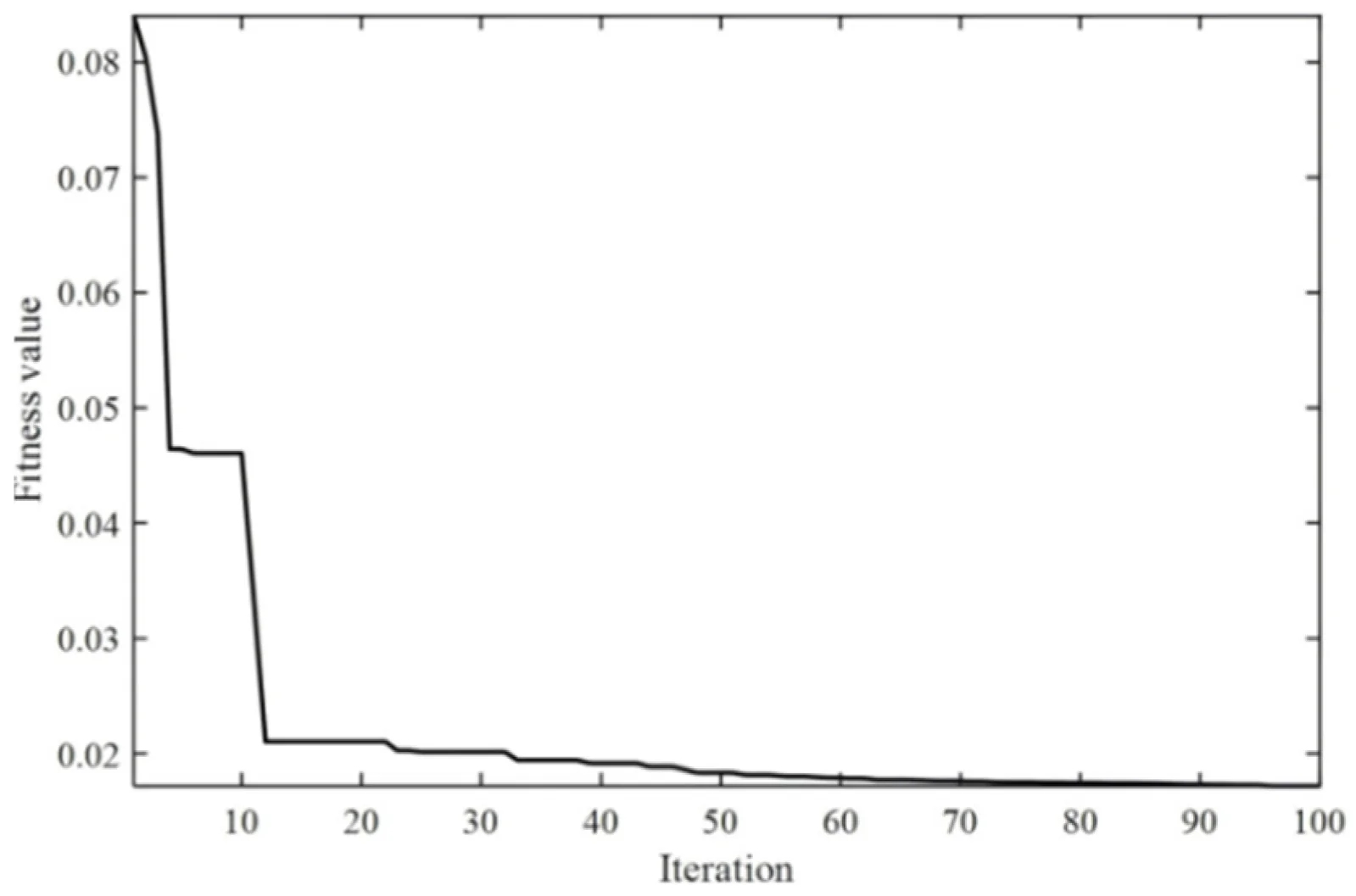
Convergence curve of MSKET-BP.

The convergence curve of MSKET-BP is shown in Figure 25. The fitness value decreases rapidly in the early stage and gradually becomes stable in the later stage, indicating good convergence behaviour during model optimization.

Overall, the results in Table 13 and Figure 23, Figure 24 and Figure 25 demonstrate that MSKET-BP achieves good prediction accuracy and stability in predicting the active component content of white-flowered *Peucedanum praeruptorum*. These results suggest that using MSKET to optimize the initial parameters of the BP neural network can effectively improve near-infrared spectral regression modelling and provide a feasible method for the rapid prediction of the total content of praeruptorin A and praeruptorin B.

## 5. Conclusions

This study developed a Multi-Strategy Kangaroo Escape Optimization Technique (MSKET) by modifying four components of the original KET search process. Adaptive stage switching and candidate-group guidance were introduced for stage allocation and safe-area selection, while greedy DE/rand-to-best/1 refinement and beta-distribution opposition-based learning were assigned to low-ranked individuals and global-best updating, respectively. The contribution lies in the KET-specific coordination of these established mechanisms.

On the 100-dimensional benchmark suites, MSKET ranked first on 22 of 29 CEC2017 functions and 8 of 10 CEC2020 functions. The ablation, diversity, and nonparametric statistical analyses showed that the combined mechanisms produced stronger overall performance than the individual variants. MSKET also achieved the best overall repeated-run performance on the piston–lever and three-bar truss design problems. In the NIR application, MSKET-BP obtained an R^2^ of 0.86440, an RMSE of 2.1377, and a MAPE of 5.0083% on the testing set.

These findings indicate that MSKET improved the search behaviour of KET within the examined benchmark, engineering, and regression tasks. However, the improvement was accompanied by higher function evaluation and computational costs. In addition, the benchmark comparison used an equal-iteration budget, and the NIR results were based on a single data split. Future work will therefore focus on reducing computational cost, conducting equal-evaluation comparisons, and validating the method on additional engineering problems and independent spectral datasets.

## Figures and Tables

**Table 1:** Comparison of recent improved metaheuristic algorithms and MSKET.

Method	Base Optimizer	Algorithm-Specific Design	Author-Reported Issue	Validation
MSEFA [8]	Firefly algorithm	Priority-roulette selection among three search strategies and adaptive control of step-size decay	Poor coordination between exploration and exploitation and inflexible step-size control	CEC2013, CEC2019, and four engineering problems
AGSMA [9]	Slime Mould Algorithm	Two subpopulations with average-fitness-based size adjustment, a new search mechanism, and a learning operator	Poor diversity, slow convergence, and premature convergence	Thirteen standard functions, CEC2021, and engineering problems
ABetaCOBL [10]	Differential evolution	Adaptation of the beta-opposition search space according to the current population distribution before opposite solutions are generated	An opposition search space that does not adapt to the current population distribution	CEC2017 and CEC2011 using one classical and two advanced DE variants
ISO [11]	Snake optimizer	A multi-strategy chaotic system for initialization, an anti-predator strategy for exploration, and bidirectional population evolution dynamics for exploitation	Low population diversity and insufficient optimization accuracy	120 numerical problems, including CEC2017 and CEC2022, eight engineering problems, and two NP-hard problems
MSKET	Kangaroo Escape Optimization Technique	Adaptive stage switching, population-proportion-based candidate guidance, selective greedy DE/rand-to-best/1 refinement, and beta-distribution opposition-based guidance	Rigid stage transition, restricted safe-area guidance, insufficient refinement of low-ranked individuals, and limited use of population-state and global-best information	CEC2017, CEC2020, constrained engineering design, and NIR prediction

**Table 2:** Algorithm-specific parameter settings of MSKET.

Parameter	Setting	Parameter	Setting
Stage 1 selection probability $p_{s}^{t}$	0.8 − 0.5 *t*/*T*	Candidate-group size $K_{s}$	max(1,round(0.30N))
Long-jump energy threshold	0.5	Candidate-group selection probability	0.75
Initial chaotic factor $\chi^{1}$	0.7	Individuals selected for DE refinement	$\mathrm{rank}(i)\geq \left\lceil 0.7N\right\rceil$
Logistic-map coefficient	4	Differential scaling factor $F_{i}^{t}$	$F_{i}^{t}\sim U(0,1)$
Zigzag perturbation coefficient $\beta_{z}$	1	BD-OBL mode-selection probability	0.5 for each mode
Maximum deflection angle $\theta_{\max}$	30°	Gaussian perturbation in BD-OBL $\eta^{t}$	$\eta^{t}\sim N(0,0.5^{2})$
DE mutation trigger probability	0.5	Long-jump stochastic trigger probability	0.5

**Table 3:** Statistical results of CEC2017 test functions F1 and F3–F10.

*f*	Results	MSKET	KET	NDO	ESC	DBA	ALA	HHO	PSO	SMA	UFIA
*F*_1_	Min	1.55 × 10^4^	1.23 × 10^8^	4.66 × 10^8^	3.39 × 10^10^	1.07 × 10^6^	1.38 × 10^9^	4.92 × 10^9^	3.49 × 10^10^	2.87 × 10^7^	2.35 × 10^10^
	Std	6.51 × 10^4^	6.96 × 10^7^	3.97 × 10^9^	1.01 × 10^10^	3.62 × 10^5^	1.27 × 10^9^	1.81 × 10^9^	1.30 × 10^10^	1.80 × 10^7^	1.18 × 10^10^
	Avg	5.66 × 10^4^	2.31 × 10^8^	5.94 × 10^9^	4.71 × 10^10^	1.62 × 10^6^	2.83 × 10^9^	8.18 × 10^9^	6.08 × 10^10^	5.83 × 10^7^	5.59 × 10^10^
	Rank	1	4	6	8	2	5	7	10	3	9
*F*_3_	Min	1.11 × 10^5^	2.23 × 10^5^	4.29 × 10^5^	3.53 × 10^5^	1.72 × 10^5^	1.67 × 10^5^	2.91 × 10^5^	4.03 × 10^5^	2.02 × 10^5^	2.60 × 10^5^
	Std	2.52 × 10^4^	8.00 × 10^4^	5.81 × 10^4^	7.11 × 10^4^	1.25 × 10^4^	4.05 × 10^4^	1.25 × 10^4^	1.06 × 10^5^	1.91 × 10^5^	3.88 × 10^4^
	Avg	1.49 × 10^5^	3.75 × 10^5^	5.16 × 10^5^	5.15 × 10^5^	2.00 × 10^5^	2.62 × 10^5^	3.18 × 10^5^	5.30 × 10^5^	4.92 × 10^5^	3.65 × 10^5^
	Rank	1	6	9	8	2	3	4	10	7	5
*F*_4_	Min	6.82 × 10^2^	9.45 × 10^2^	9.21 × 10^2^	3.62 × 10^3^	6.78 × 10^2^	9.92 × 10^2^	1.83 × 10^3^	3.64 × 10^3^	7.12 × 10^2^	3.53 × 10^3^
	Std	5.76 × 10^1^	1.05 × 10^2^	3.83 × 10^2^	1.99 × 10^3^	5.61 × 10^1^	1.82 × 10^2^	5.16 × 10^2^	3.07 × 10^3^	6.27 × 10^1^	2.69 × 10^3^
	Avg	8.14 × 10^2^	1.13 × 10^3^	1.47 × 10^3^	6.12 × 10^3^	7.69 × 10^2^	1.28 × 10^3^	2.63 × 10^3^	8.90 × 10^3^	8.44 × 10^2^	8.24 × 10^3^
	Rank	2	4	6	8	1	5	7	10	3	9
*F*_5_	Min	9.08 × 10^2^	1.15 × 10^3^	9.54 × 10^2^	8.41 × 10^2^	1.24 × 10^3^	1.03 × 10^3^	1.48 × 10^3^	1.31 × 10^3^	1.10 × 10^3^	1.35 × 10^3^
	Std	6.86 × 10^1^	7.49 × 10^1^	8.34 × 10^1^	9.55 × 10^1^	3.94 × 10^1^	9.11 × 10^1^	5.03 × 10^1^	8.74 × 10^1^	9.71 × 10^1^	1.35 × 10^2^
	Avg	1.03 × 10^3^	1.28 × 10^3^	1.11 × 10^3^	9.96 × 10^2^	1.33 × 10^3^	1.20 × 10^3^	1.60 × 10^3^	1.47 × 10^3^	1.29 × 10^3^	1.67 × 10^3^
	Rank	2	5	3	1	7	4	9	8	6	10
*F*_6_	Min	6.25 × 10^2^	6.48 × 10^2^	6.19 × 10^2^	6.14 × 10^2^	6.64 × 10^2^	6.39 × 10^2^	6.78 × 10^2^	6.59 × 10^2^	6.44 × 10^2^	6.78 × 10^2^
	Std	7.68 × 10^0^	5.87 × 10^0^	1.34 × 10^1^	3.14 × 10^0^	2.08 × 10^0^	7.30 × 10^0^	4.31 × 10^0^	5.64 × 10^0^	6.00 × 10^0^	5.57 × 10^0^
	Avg	6.38 × 10^2^	6.59 × 10^2^	6.37 × 10^2^	6.20 × 10^2^	6.67 × 10^2^	6.52 × 10^2^	6.86 × 10^2^	6.70 × 10^2^	6.57 × 10^2^	6.91 × 10^2^
	Rank	3	6	2	1	7	4	9	8	5	10
*F*_7_	Min	1.47 × 10^3^	2.28 × 10^3^	1.87 × 10^3^	1.55 × 10^3^	2.97 × 10^3^	1.75 × 10^3^	3.49 × 10^3^	2.44 × 10^3^	1.85 × 10^3^	2.97 × 10^3^
	Std	1.42 × 10^2^	5.13 × 10^2^	2.23 × 10^2^	1.45 × 10^2^	8.19 × 10^1^	1.69 × 10^2^	1.10 × 10^2^	3.03 × 10^2^	1.98 × 10^2^	1.85 × 10^2^
	Avg	1.72 × 10^3^	3.12 × 10^3^	2.25 × 10^3^	1.80 × 10^3^	3.18 × 10^3^	2.20 × 10^3^	3.75 × 10^3^	3.16 × 10^3^	2.11 × 10^3^	3.37 × 10^3^
	Rank	1	6	5	2	8	4	10	7	3	9
*F*_8_	Min	1.19 × 10^3^	1.40 × 10^3^	1.25 × 10^3^	1.20 × 10^3^	1.68 × 10^3^	1.32 × 10^3^	1.92 × 10^3^	1.65 × 10^3^	1.43 × 10^3^	1.81 × 10^3^
	Std	6.44 × 10^1^	1.33 × 10^2^	7.97 × 10^1^	7.52 × 10^1^	5.64 × 10^1^	9.62 × 10^1^	6.44 × 10^1^	1.02 × 10^2^	1.23 × 10^2^	1.71 × 10^2^
	Avg	1.32 × 10^3^	1.65 × 10^3^	1.42 × 10^3^	1.33 × 10^3^	1.80 × 10^3^	1.45 × 10^3^	2.06 × 10^3^	1.86 × 10^3^	1.64 × 10^3^	2.10 × 10^3^
	Rank	1	6	3	2	7	4	9	8	5	10
*F*_9_	Min	9.89 × 10^3^	1.85 × 10^4^	9.44 × 10^3^	1.09 × 10^4^	2.50 × 10^4^	2.18 × 10^4^	5.61 × 10^4^	2.28 × 10^4^	2.84 × 10^4^	4.44 × 10^4^
	Std	2.71 × 10^3^	3.47 × 10^3^	1.13 × 10^4^	3.01 × 10^3^	1.08 × 10^3^	9.68 × 10^3^	3.83 × 10^3^	4.67 × 10^3^	3.49 × 10^3^	6.95 × 10^3^
	Avg	1.58 × 10^4^	2.48 × 10^4^	3.32 × 10^4^	1.66 × 10^4^	2.70 × 10^4^	3.86 × 10^4^	6.37 × 10^4^	3.08 × 10^4^	3.45 × 10^4^	5.54 × 10^4^
	Rank	1	3	6	2	4	8	10	5	7	9
*F*_10_	Min	1.25 × 10^4^	1.38 × 10^4^	1.85 × 10^4^	2.60 × 10^4^	1.34 × 10^4^	1.81 × 10^4^	1.93 × 10^4^	1.44 × 10^4^	1.44 × 10^4^	2.39 × 10^4^
	Std	1.91 × 10^3^	1.88 × 10^3^	2.79 × 10^3^	1.19 × 10^3^	1.33 × 10^3^	3.05 × 10^3^	1.93 × 10^3^	1.25 × 10^3^	1.57 × 10^3^	1.87 × 10^3^
	Avg	1.57 × 10^4^	1.70 × 10^4^	2.40 × 10^4^	2.82 × 10^4^	1.61 × 10^4^	2.46 × 10^4^	2.31 × 10^4^	1.77 × 10^4^	1.75 × 10^4^	3.02 × 10^4^
	Rank	1	3	7	9	2	8	6	5	4	10

**Table 4:** Statistical results of CEC2017 test functions F11–F20.

*f*	Results	MSKET	KET	NDO	ESC	DBA	ALA	HHO	PSO	SMA	UFIA
*F*_11_	Min	2.39 × 10^3^	8.45 × 10^3^	2.65 × 10^4^	3.92 × 10^4^	2.73 × 10^3^	1.31 × 10^4^	3.77 × 10^4^	1.79 × 10^4^	3.41 × 10^3^	2.44 × 10^4^
	Std	3.26 × 10^2^	6.88 × 10^3^	2.03 × 10^4^	1.71 × 10^4^	3.87 × 10^2^	8.65 × 10^3^	2.09 × 10^4^	1.86 × 10^4^	2.92 × 10^3^	1.61 × 10^4^
	Avg	2.99 × 10^3^	1.95 × 10^4^	6.36 × 10^4^	6.71 × 10^4^	3.35 × 10^3^	2.53 × 10^4^	6.68 × 10^4^	4.27 × 10^4^	6.40 × 10^3^	5.02 × 10^4^
	Rank	1	4	8	10	2	5	9	6	3	7
*F*_12_	Min	7.47 × 10^6^	6.86 × 10^7^	4.60 × 10^7^	1.76 × 10^9^	1.43 × 10^8^	1.05 × 10^8^	7.18 × 10^8^	3.93 × 10^9^	4.67 × 10^7^	9.22 × 10^8^
	Std	1.53 × 10^7^	8.93 × 10^7^	4.16 × 10^8^	3.06 × 10^9^	4.36 × 10^7^	1.16 × 10^8^	3.61 × 10^8^	7.51 × 10^9^	8.10 × 10^7^	3.81 × 10^9^
	Avg	3.11 × 10^7^	2.25 × 10^8^	3.49 × 10^8^	7.20 × 10^9^	2.18 × 10^8^	2.45 × 10^8^	1.35 × 10^9^	1.47 × 10^10^	2.05 × 10^8^	7.69 × 10^9^
	Rank	1	4	6	8	3	5	7	10	2	9
*F*_13_	Min	7.88 × 10^3^	2.69 × 10^4^	6.37 × 10^3^	2.97 × 10^7^	2.46 × 10^4^	2.75 × 10^4^	9.02 × 10^6^	1.34 × 10^6^	5.11 × 10^4^	9.04 × 10^5^
	Std	2.84 × 10^3^	2.05 × 10^4^	6.91 × 10^3^	6.40 × 10^8^	1.35 × 10^4^	4.88 × 10^6^	6.92 × 10^6^	9.32 × 10^8^	1.00 × 10^6^	6.22 × 10^8^
	Avg	1.13 × 10^4^	5.42 × 10^4^	1.52 × 10^4^	4.83 × 10^8^	4.01 × 10^4^	9.42 × 10^5^	1.87 × 10^7^	7.57 × 10^8^	4.81 × 10^5^	2.39 × 10^8^
	Rank	1	4	2	9	3	6	7	10	5	8
*F*_14_	Min	1.75 × 10^5^	5.10 × 10^5^	2.38 × 10^5^	1.00 × 10^6^	6.38 × 10^5^	4.54 × 10^4^	3.20 × 10^6^	5.37 × 10^5^	7.33 × 10^5^	3.25 × 10^5^
	Std	2.91 × 10^5^	9.91 × 10^5^	1.13 × 10^6^	3.58 × 10^6^	2.10 × 10^5^	3.98 × 10^5^	1.57 × 10^6^	2.65 × 10^6^	1.88 × 10^6^	1.60 × 10^6^
	Avg	5.39 × 10^5^	1.83 × 10^6^	2.11 × 10^6^	7.12 × 10^6^	9.52 × 10^5^	4.77 × 10^5^	6.06 × 10^6^	3.85 × 10^6^	3.10 × 10^6^	1.56 × 10^6^
	Rank	2	5	6	10	3	1	9	8	7	4
*F*_15_	Min	2.28 × 10^3^	1.10 × 10^4^	2.31 × 10^3^	1.64 × 10^4^	1.44 × 10^4^	2.84 × 10^3^	2.27 × 10^6^	2.90 × 10^4^	2.85 × 10^4^	2.07 × 10^4^
	Std	2.22 × 10^3^	1.48 × 10^4^	3.33 × 10^3^	8.56 × 10^7^	1.44 × 10^4^	1.61 × 10^4^	2.95 × 10^6^	5.82 × 10^8^	4.58 × 10^5^	4.73 × 10^6^
	Avg	4.07 × 10^3^	3.12 × 10^4^	5.51 × 10^3^	2.28 × 10^7^	3.08 × 10^4^	1.25 × 10^4^	4.93 × 10^6^	3.57 × 10^8^	2.92 × 10^5^	1.32 × 10^6^
	Rank	1	5	2	9	4	3	8	10	6	7
*F*_16_	Min	3.80 × 10^3^	5.02 × 10^3^	4.29 × 10^3^	4.39 × 10^3^	5.07 × 10^3^	4.85 × 10^3^	6.82 × 10^3^	5.29 × 10^3^	4.96 × 10^3^	5.99 × 10^3^
	Std	6.48 × 10^2^	5.32 × 10^2^	8.87 × 10^2^	9.76 × 10^2^	9.00 × 10^2^	7.67 × 10^2^	9.33 × 10^2^	8.67 × 10^2^	6.55 × 10^2^	1.20 × 10^3^
	Avg	5.31 × 10^3^	6.09 × 10^3^	5.87 × 10^3^	6.24 × 10^3^	7.34 × 10^3^	6.32 × 10^3^	8.64 × 10^3^	7.29 × 10^3^	6.29 × 10^3^	7.33 × 10^3^
	Rank	1	3	2	4	9	6	10	7	5	8
*F*_17_	Min	3.95 × 10^3^	4.64 × 10^3^	3.94 × 10^3^	3.72 × 10^3^	3.77 × 10^3^	4.28 × 10^3^	5.90 × 10^3^	5.23 × 10^3^	4.52 × 10^3^	5.68 × 10^3^
	Std	3.89 × 10^2^	5.26 × 10^2^	5.70 × 10^2^	6.23 × 10^2^	6.32 × 10^2^	6.69 × 10^2^	6.24 × 10^2^	1.42 × 10^3^	5.93 × 10^2^	2.78 × 10^3^
	Avg	4.76 × 10^3^	5.57 × 10^3^	4.98 × 10^3^	4.96 × 10^3^	5.78 × 10^3^	5.58 × 10^3^	7.19 × 10^3^	6.57 × 10^3^	5.67 × 10^3^	8.29 × 10^3^
	Rank	1	4	3	2	7	5	9	8	6	10
*F*_18_	Min	3.25 × 10^5^	6.68 × 10^5^	1.52 × 10^6^	2.77 × 10^6^	8.93 × 10^5^	2.04 × 10^5^	2.17 × 10^6^	7.56 × 10^5^	1.63 × 10^6^	6.10 × 10^5^
	Std	3.21 × 10^5^	1.17 × 10^6^	2.89 × 10^6^	5.47 × 10^6^	3.17 × 10^5^	5.77 × 10^5^	2.80 × 10^6^	2.32 × 10^6^	2.97 × 10^6^	1.34 × 10^6^
	Avg	7.73 × 10^5^	2.44 × 10^6^	4.93 × 10^6^	8.48 × 10^6^	1.64 × 10^6^	9.87 × 10^5^	6.58 × 10^6^	4.00 × 10^6^	8.22 × 10^6^	3.02 × 10^6^
	Rank	1	4	7	10	3	2	8	6	9	5
*F*_19_	Min	2.32 × 10^3^	1.50 × 10^4^	2.12 × 10^3^	1.00 × 10^5^	2.95 × 10^5^	2.58 × 10^3^	5.01 × 10^6^	9.10 × 10^5^	2.81 × 10^4^	2.79 × 10^4^
	Std	4.82 × 10^3^	7.38 × 10^4^	4.22 × 10^3^	5.74 × 10^7^	1.58 × 10^6^	2.90 × 10^4^	5.20 × 10^6^	7.61 × 10^8^	1.26 × 10^5^	2.62 × 10^7^
	Avg	5.38 × 10^3^	1.08 × 10^5^	5.70 × 10^3^	3.33 × 10^7^	2.50 × 10^6^	1.72 × 10^4^	1.35 × 10^7^	4.38 × 10^8^	1.75 × 10^5^	8.21 × 10^6^
	Rank	1	4	2	9	6	3	8	10	5	7
*F*_20_	Min	4.30 × 10^3^	4.64 × 10^3^	5.15 × 10^3^	4.87 × 10^3^	4.49 × 10^3^	5.28 × 10^3^	5.17 × 10^3^	4.35 × 10^3^	4.59 × 10^3^	6.11 × 10^3^
	Std	4.33 × 10^2^	5.03 × 10^2^	6.07 × 10^2^	4.33 × 10^2^	6.72 × 10^2^	5.84 × 10^2^	5.36 × 10^2^	5.97 × 10^2^	5.48 × 10^2^	4.09 × 10^2^
	Avg	5.13 × 10^3^	5.61 × 10^3^	6.08 × 10^3^	5.84 × 10^3^	5.83 × 10^3^	6.15 × 10^3^	6.12 × 10^3^	5.48 × 10^3^	5.56 × 10^3^	7.02 × 10^3^
	Rank	1	4	7	6	5	9	8	2	3	10

**Table 5:** Statistical results of CEC2017 test functions F21–F30.

*f*	Results	MSKET	KET	NDO	ESC	DBA	ALA	HHO	PSO	SMA	UFIA
*F*_21_	Min	2.60 × 10^3^	2.93 × 10^3^	2.79 × 10^3^	2.70 × 10^3^	3.62 × 10^3^	2.83 × 10^3^	3.81 × 10^3^	3.41 × 10^3^	2.92 × 10^3^	3.35 × 10^3^
	Std	5.69 × 10^1^	1.16 × 10^2^	9.39 × 10^1^	1.13 × 10^2^	2.37 × 10^2^	7.71 × 10^1^	2.06 × 10^2^	1.95 × 10^2^	1.07 × 10^2^	2.09 × 10^2^
	Avg	2.75 × 10^3^	3.16 × 10^3^	2.96 × 10^3^	2.85 × 10^3^	4.06 × 10^3^	3.03 × 10^3^	4.26 × 10^3^	3.69 × 10^3^	3.11 × 10^3^	3.74 × 10^3^
	Rank	1	6	3	2	9	4	10	7	5	8
*F*_22_	Min	1.57 × 10^4^	1.66 × 10^4^	1.93 × 10^4^	2.75 × 10^4^	1.94 × 10^4^	2.04 × 10^4^	2.38 × 10^4^	1.89 × 10^4^	1.76 × 10^4^	2.81 × 10^4^
	Std	1.48 × 10^3^	2.05 × 10^3^	3.02 × 10^3^	9.93 × 10^2^	1.03 × 10^3^	2.94 × 10^3^	1.22 × 10^3^	1.22 × 10^3^	1.49 × 10^3^	1.79 × 10^3^
	Avg	1.97 × 10^4^	2.03 × 10^4^	2.57 × 10^4^	3.01 × 10^4^	2.13 × 10^4^	2.63 × 10^4^	2.64 × 10^4^	2.12 × 10^4^	1.98 × 10^4^	3.29 × 10^4^
	Rank	1	3	6	9	5	7	8	4	2	10
*F*_23_	Min	3.15 × 10^3^	3.48 × 10^3^	3.30 × 10^3^	3.24 × 10^3^	5.47 × 10^3^	3.32 × 10^3^	4.88 × 10^3^	4.48 × 10^3^	3.29 × 10^3^	4.33 × 10^3^
	Std	1.05 × 10^2^	1.87 × 10^2^	1.17 × 10^2^	5.67 × 10^1^	3.09 × 10^2^	1.07 × 10^2^	4.27 × 10^2^	3.49 × 10^2^	9.00 × 10^1^	2.23 × 10^2^
	Avg	3.37 × 10^3^	3.74 × 10^3^	3.55 × 10^3^	3.38 × 10^3^	5.95 × 10^3^	3.54 × 10^3^	5.54 × 10^3^	4.98 × 10^3^	3.47 × 10^3^	4.69 × 10^3^
	Rank	1	6	5	2	10	4	9	8	3	7
*F*_24_	Min	3.77 × 10^3^	3.99 × 10^3^	3.98 × 10^3^	3.97 × 10^3^	5.58 × 10^3^	3.88 × 10^3^	6.70 × 10^3^	5.45 × 10^3^	3.88 × 10^3^	5.53 × 10^3^
	Std	1.17 × 10^2^	2.14 × 10^2^	2.10 × 10^2^	1.10 × 10^2^	3.63 × 10^2^	2.51 × 10^2^	4.59 × 10^2^	5.00 × 10^2^	1.24 × 10^2^	4.73 × 10^2^
	Avg	3.94 × 10^3^	4.36 × 10^3^	4.35 × 10^3^	4.12 × 10^3^	6.11 × 10^3^	4.35 × 10^3^	7.46 × 10^3^	6.16 × 10^3^	4.16 × 10^3^	6.34 × 10^3^
	Rank	1	6	5	2	7	4	10	8	3	9
*F*_25_	Min	3.33 × 10^3^	3.61 × 10^3^	3.71 × 10^3^	6.01 × 10^3^	3.31 × 10^3^	3.84 × 10^3^	4.18 × 10^3^	5.74 × 10^3^	3.43 × 10^3^	5.91 × 10^3^
	Std	5.94 × 10^1^	1.05 × 10^2^	3.86 × 10^2^	9.37 × 10^2^	5.65 × 10^1^	1.34 × 10^2^	2.27 × 10^2^	1.33 × 10^3^	6.27 × 10^1^	1.33 × 10^3^
	Avg	3.45 × 10^3^	3.81 × 10^3^	4.22 × 10^3^	7.45 × 10^3^	3.44 × 10^3^	4.09 × 10^3^	4.57 × 10^3^	8.24 × 10^3^	3.55 × 10^3^	7.88 × 10^3^
	Rank	2	4	6	8	1	5	7	10	3	9
*F*_26_	Min	1.09 × 10^4^	1.50 × 10^4^	1.29 × 10^4^	1.18 × 10^4^	1.98 × 10^4^	1.24 × 10^4^	2.65 × 10^4^	1.44 × 10^4^	4.22 × 10^3^	2.56 × 10^4^
	Std	1.07 × 10^3^	2.51 × 10^3^	2.51 × 10^3^	1.97 × 10^3^	2.45 × 10^3^	1.50 × 10^3^	1.61 × 10^3^	4.63 × 10^3^	2.79 × 10^3^	2.53 × 10^3^
	Avg	1.27 × 10^4^	1.88 × 10^4^	1.73 × 10^4^	1.53 × 10^4^	2.64 × 10^4^	1.50 × 10^4^	2.92 × 10^4^	2.73 × 10^4^	1.42 × 10^4^	3.10 × 10^4^
	Rank	1	6	5	4	7	3	9	8	2	10
*F*_27_	Min	3.54 × 10^3^	3.81 × 10^3^	3.69 × 10^3^	3.77 × 10^3^	6.89 × 10^3^	3.54 × 10^3^	4.22 × 10^3^	3.97 × 10^3^	3.49 × 10^3^	4.45 × 10^3^
	Std	1.26 × 10^2^	2.36 × 10^2^	2.13 × 10^2^	1.58 × 10^2^	1.37 × 10^3^	9.93 × 10^1^	6.00 × 10^2^	3.24 × 10^2^	1.04 × 10^2^	7.36 × 10^2^
	Avg	3.73 × 10^3^	4.09 × 10^3^	4.09 × 10^3^	4.09 × 10^3^	9.48 × 10^3^	3.69 × 10^3^	5.23 × 10^3^	4.56 × 10^3^	3.66 × 10^3^	5.56 × 10^3^
	Rank	3	6	5	4	10	2	8	7	1	9
*F*_28_	Min	3.47 × 10^3^	3.89 × 10^3^	4.11 × 10^3^	8.94 × 10^3^	3.44 × 10^3^	3.98 × 10^3^	4.74 × 10^3^	7.55 × 10^3^	3.54 × 10^3^	7.33 × 10^3^
	Std	3.99 × 10^1^	2.09 × 10^2^	8.00 × 10^2^	1.71 × 10^3^	5.19 × 10^1^	5.75 × 10^3^	4.85 × 10^2^	1.95 × 10^3^	3.57 × 10^1^	1.18 × 10^3^
	Avg	3.56 × 10^3^	4.13 × 10^3^	5.09 × 10^3^	1.13 × 10^4^	3.53 × 10^3^	7.73 × 10^3^	5.68 × 10^3^	1.12 × 10^4^	3.59 × 10^3^	9.64 × 10^3^
	Rank	2	4	5	10	1	7	6	9	3	8
*F*_29_	Min	5.58 × 10^3^	6.86 × 10^3^	6.22 × 10^3^	6.18 × 10^3^	9.22 × 10^3^	6.00 × 10^3^	9.19 × 10^3^	8.00 × 10^3^	6.08 × 10^3^	8.86 × 10^3^
	Std	5.61 × 10^2^	6.39 × 10^2^	5.99 × 10^2^	6.11 × 10^2^	9.10 × 10^2^	7.89 × 10^2^	1.02 × 10^3^	1.19 × 10^3^	7.10 × 10^2^	1.19 × 10^3^
	Avg	7.04 × 10^3^	8.18 × 10^3^	7.28 × 10^3^	7.38 × 10^3^	1.08 × 10^4^	7.40 × 10^3^	1.11 × 10^4^	1.03 × 10^4^	7.67 × 10^3^	1.09 × 10^4^
	Rank	1	6	2	3	8	4	10	7	5	9
*F*_30_	Min	2.23 × 10^4^	1.00 × 10^6^	8.10 × 10^4^	2.53 × 10^7^	9.66 × 10^6^	7.86 × 10^4^	6.82 × 10^7^	3.07 × 10^7^	1.43 × 10^6^	4.76 × 10^6^
	Std	6.36 × 10^4^	2.15 × 10^6^	4.80 × 10^6^	2.81 × 10^8^	6.20 × 10^6^	2.65 × 10^5^	6.91 × 10^7^	1.10 × 10^9^	2.17 × 10^6^	8.01 × 10^8^
	Avg	1.17 × 10^5^	4.37 × 10^6^	3.55 × 10^6^	3.22 × 10^8^	2.00 × 10^7^	3.59 × 10^5^	1.66 × 10^8^	1.07 × 10^9^	4.88 × 10^6^	4.25 × 10^8^
	Rank	1	4	3	8	6	2	7	10	5	9

**Table 6:** Results of the Wilcoxon rank-sum test for CEC2017.

Compared Algorithms	+/−/=	Final Rank
MSKET vs. KET	28/0/1	MSKET (1)
MSKET vs. NDO	25/0/4	SMA (2)
MSKET vs. ESC	22/1/6	ALA (3)
MSKET vs. DBA	25/2/2	KET (4)
MSKET vs. ALA	25/0/4	NDO (5)
MSKET vs. HHO	29/0/0	DBA (6)
MSKET vs. PSO	29/0/0	ESC (7)
MSKET vs. SMA	26/1/2	PSO (8)
MSKET vs. UFIA	29/0/0	HHO (9)
		UFIA (10)

**Table 7:** Statistical results of CEC2020 test functions F1–F10.

*f*	Results	MSKET	KET	NDO	ESC	DBA	ALA	HHO	PSO	SMA	UFIA
*F*_1_	Min	1.12 × 10^4^	9.87 × 10^7^	1.18 × 10^9^	2.75 × 10^10^	8.26 × 10^5^	1.54 × 10^9^	4.89 × 10^9^	4.15 × 10^10^	3.97 × 10^7^	2.95 × 10^10^
	Std	2.33 × 10^4^	9.49 × 10^7^	2.65 × 10^9^	1.13 × 10^10^	3.86 × 10^5^	7.13 × 10^8^	1.71 × 10^9^	1.64 × 10^10^	1.18 × 10^7^	1.08 × 10^10^
	Avg	3.83 × 10^4^	2.41 × 10^8^	4.21 × 10^9^	4.92 × 10^10^	1.55 × 10^6^	2.53 × 10^9^	7.66 × 10^9^	6.81 × 10^10^	5.67 × 10^7^	5.78 × 10^10^
	Rank	1	4	6	8	2	5	7	10	3	9
*F*_2_	Min	1.42 × 10^4^	1.45 × 10^4^	1.86 × 10^4^	2.57 × 10^4^	1.44 × 10^4^	1.97 × 10^4^	1.99 × 10^4^	1.43 × 10^4^	1.57 × 10^4^	2.65 × 10^4^
	Std	1.53 × 10^3^	1.83 × 10^3^	2.42 × 10^3^	1.09 × 10^3^	1.34 × 10^3^	2.40 × 10^3^	1.47 × 10^3^	1.47 × 10^3^	1.36 × 10^3^	1.70 × 10^3^
	Avg	1.64 × 10^4^	1.73 × 10^4^	2.34 × 10^4^	2.83 × 10^4^	1.67 × 10^4^	2.49 × 10^4^	2.32 × 10^4^	1.78 × 10^4^	1.79 × 10^4^	3.06 × 10^4^
	Rank	1	3	7	9	2	8	6	4	5	10
*F*_3_	Min	1.52 × 10^3^	2.27 × 10^3^	1.83 × 10^3^	1.37 × 10^3^	2.82 × 10^3^	1.81 × 10^3^	3.39 × 10^3^	2.64 × 10^3^	1.70 × 10^3^	2.77 × 10^3^
	Std	1.24 × 10^2^	3.99 × 10^2^	2.52 × 10^2^	2.25 × 10^2^	1.05 × 10^2^	2.06 × 10^2^	1.33 × 10^2^	2.86 × 10^2^	2.25 × 10^2^	2.24 × 10^2^
	Avg	1.74 × 10^3^	2.99 × 10^3^	2.23 × 10^3^	1.75 × 10^3^	3.13 × 10^3^	2.21 × 10^3^	3.76 × 10^3^	3.20 × 10^3^	2.10 × 10^3^	3.43 × 10^3^
	Rank	1	6	5	2	7	4	10	8	3	9
*F*_4_	Min	2.00 × 10^3^	2.10 × 10^3^	2.40 × 10^3^	1.10 × 10^4^	2.12 × 10^3^	2.54 × 10^3^	3.45 × 10^3^	8.49 × 10^4^	2.01 × 10^3^	5.74 × 10^4^
	Std	4.26 × 10^1^	1.53 × 10^2^	4.05 × 10^3^	7.00 × 10^4^	4.16 × 10^1^	1.12 × 10^3^	1.03 × 10^4^	3.79 × 10^5^	4.19 × 10^1^	1.95 × 10^5^
	Avg	2.06 × 10^3^	2.35 × 10^3^	5.05 × 10^3^	9.12 × 10^4^	2.19 × 10^3^	3.87 × 10^3^	1.73 × 10^4^	5.76 × 10^5^	2.06 × 10^3^	3.34 × 10^5^
	Rank	1	4	6	8	3	5	7	10	2	9
*F*_5_	Min	1.64 × 10^6^	5.83 × 10^6^	4.86 × 10^6^	3.25 × 10^7^	2.12 × 10^6^	7.11 × 10^6^	3.18 × 10^7^	1.32 × 10^7^	1.12 × 10^7^	7.72 × 10^6^
	Std	1.32 × 10^6^	7.66 × 10^6^	6.81 × 10^6^	3.12 × 10^7^	9.00 × 10^5^	9.41 × 10^6^	2.26 × 10^7^	2.43 × 10^7^	7.77 × 10^6^	1.51 × 10^7^
	Avg	3.79 × 10^6^	1.53 × 10^7^	1.36 × 10^7^	7.00 × 10^7^	3.61 × 10^6^	2.11 × 10^7^	6.20 × 10^7^	4.81 × 10^7^	2.11 × 10^7^	3.00 × 10^7^
	Rank	2	4	3	10	1	6	9	8	5	7
*F*_6_	Min	4.82 × 10^3^	5.40 × 10^3^	4.25 × 10^3^	2.67 × 10^3^	7.19 × 10^3^	5.35 × 10^3^	8.88 × 10^3^	5.93 × 10^3^	5.71 × 10^3^	7.58 × 10^3^
	Std	6.31 × 10^2^	9.85 × 10^2^	8.22 × 10^2^	7.40 × 10^2^	1.11 × 10^3^	1.02 × 10^3^	1.21 × 10^3^	1.24 × 10^3^	5.89 × 10^2^	1.10 × 10^3^
	Avg	5.91 × 10^3^	6.98 × 10^3^	5.85 × 10^3^	4.74 × 10^3^	8.86 × 10^3^	7.38 × 10^3^	1.08 × 10^4^	8.36 × 10^3^	6.62 × 10^3^	9.81 × 10^3^
	Rank	3	5	2	1	8	6	10	7	4	9
*F*_7_	Min	7.59 × 10^5^	3.01 × 10^6^	1.95 × 10^6^	8.56 × 10^6^	1.88 × 10^6^	2.40 × 10^6^	6.21 × 10^6^	3.08 × 10^6^	2.92 × 10^6^	4.33 × 10^6^
	Std	1.06 × 10^6^	3.13 × 10^6^	2.40 × 10^6^	1.03 × 10^7^	5.73 × 10^5^	3.76 × 10^6^	8.82 × 10^6^	8.03 × 10^6^	3.17 × 10^6^	6.26 × 10^6^
	Avg	2.33 × 10^6^	6.68 × 10^6^	5.72 × 10^6^	2.28 × 10^7^	3.03 × 10^6^	7.51 × 10^6^	2.14 × 10^7^	1.49 × 10^7^	7.68 × 10^6^	1.17 × 10^7^
	Rank	1	4	3	10	2	5	9	8	6	7
*F*_8_	Min	1.51 × 10^4^	1.70 × 10^4^	2.14 × 10^4^	2.67 × 10^4^	1.95 × 10^4^	2.11 × 10^4^	2.31 × 10^4^	1.76 × 10^4^	1.76 × 10^4^	2.96 × 10^4^
	Std	1.90 × 10^3^	1.83 × 10^3^	2.27 × 10^3^	1.12 × 10^3^	1.06 × 10^3^	2.50 × 10^3^	1.19 × 10^3^	1.42 × 10^3^	1.11 × 10^3^	1.35 × 10^3^
	Avg	1.89 × 10^4^	1.99 × 10^4^	2.56 × 10^4^	2.98 × 10^4^	2.08 × 10^4^	2.62 × 10^4^	2.54 × 10^4^	2.13 × 10^4^	1.99 × 10^4^	3.33 × 10^4^
	Rank	1	3	7	9	4	8	6	5	2	10
*F*_9_	Min	3.77 × 10^3^	4.04 × 10^3^	3.98 × 10^3^	3.90 × 10^3^	5.35 × 10^3^	3.89 × 10^3^	6.70 × 10^3^	4.99 × 10^3^	3.85 × 10^3^	5.29 × 10^3^
	Std	9.88 × 10^1^	1.61 × 10^2^	1.67 × 10^2^	1.09 × 10^2^	4.56 × 10^2^	1.84 × 10^2^	5.22 × 10^2^	5.32 × 10^2^	1.43 × 10^2^	4.90 × 10^2^
	Avg	3.91 × 10^3^	4.43 × 10^3^	4.35 × 10^3^	4.08 × 10^3^	6.23 × 10^3^	4.31 × 10^3^	7.52 × 10^3^	6.21 × 10^3^	4.10 × 10^3^	6.01 × 10^3^
	Rank	1	6	5	2	9	4	10	8	3	7
*F*_10_	Min	3.38 × 10^3^	3.64 × 10^3^	3.58 × 10^3^	5.18 × 10^3^	3.34 × 10^3^	3.81 × 10^3^	4.22 × 10^3^	6.24 × 10^3^	3.42 × 10^3^	5.84 × 10^3^
	Std	4.82 × 10^1^	8.43 × 10^1^	2.92 × 10^2^	1.37 × 10^3^	4.22 × 10^1^	1.26 × 10^2^	2.17 × 10^2^	9.31 × 10^2^	5.89 × 10^1^	1.04 × 10^3^
	Avg	3.46 × 10^3^	3.77 × 10^3^	4.23 × 10^3^	7.56 × 10^3^	3.46 × 10^3^	4.05 × 10^3^	4.56 × 10^3^	7.58 × 10^3^	3.53 × 10^3^	7.50 × 10^3^
	Rank	1	4	6	9	2	5	7	10	3	8

**Table 8:** Results of the Wilcoxon rank-sum test for CEC2020.

Compared Algorithms	+/−/=	Final Rank
MSKET vs. KET	9/0/1	MSKET (1)
MSKET vs. NDO	9/0/1	SMA (2)
MSKET vs. ESC	8/1/1	DBA (3)
MSKET vs. DBA	7/0/3	KET (4)
MSKET vs. ALA	10/0/0	NDO (5)
MSKET vs. HHO	10/0/0	ALA (6)
MSKET vs. PSO	10/0/0	ESC (7)
MSKET vs. SMA	9/0/1	PSO (8)
MSKET vs. UFIA	10/0/0	HHO (9)
		UFIA (10)

**Table 9:** Overall ablation results on CEC2017 and CEC2020.

Variant	CEC2017 Average Rank	First-Place Results	Functions Outperforming KET	CEC2020 Average Rank	First-Place Results	Functions Outperforming KET
KET	7.45	0	—	6.9	0	—
KET-AS	5.45	0	24/29	5.8	0	6/10
KET-CG	5.66	0	24/29	5.3	0	9/10
KET-DE	4.97	0	25/29	4.00	0	9/10
KET-BD	4.86	0	27/29	5.00	0	9/10
KET-AS-CG	4.03	1	29/29	4.70	0	8/10
KET-AS-CG-DE	2.52	1	29/29	3.30	0	8/10
MSKET	1.07	27	29/29	1.00	10	10/10

**Table 10:** Computational cost comparison between KET and MSKET.

Algorithm	FEs per Run	Average CPU Time (s)	Computational Complexity
KET	30,030	0.8511	*O*(*TND*)
MSKET	130,030	4.9531	$O(TN^{2}D)$

Note: The reported complexity excludes the problem-dependent objective-function evaluation cost.

**Table 11:** Statistical results of the comparison algorithms for the piston–lever design problem.

Name	MSKET	KET	NDO	ESC	DBA	ALA	HHO	PSO	SMA	UFIA
Best	1.057393888	1.057393888	1.057393888	1.057393888	1.517337204	1.057393888	1.062181174	1.057393888	1.057394252	1.057393888
Std	7.81 × 10^−14^	110.5924767	1.033543309	30.38580124	124.4973783	57.53742078	215.7650481	110.6032645	30.38315122	3.899179877
Avg	1.057393888	41.27169275	1.246092216	6.60506031	362.330203	23.24610538	295.1108672	41.27399671	6.604576762	1.840869352

**Table 12:** Statistical results of the comparison algorithms for the three-bar truss design problem.

Name	MSKET	KET	NDO	ESC	DBA	ALA	HHO	PSO	SMA	UFIA
Best	263.8958434	263.8958434	263.8958464	263.8958953	263.8958467	263.8958434	263.9039179	263.8958585	267.7255549	263.8958435
Std	1.78 × 10^−13^	0.001967724	0.021143856	0.003715184	0.007624296	9.22334 × 10^−7^	0.094099088	0.01414834	1.333172573	0.000210286
Avg	263.8958434	263.8963485	263.9166802	263.8986275	263.9007359	263.8958439	264.0146675	263.9024987	271.0454594	263.8960178

**Table 13:** Prediction performance comparison of different models on the testing set.

Model	*R* ^2^	RMSE	MAPE (%)
BP	0.61992	3.5789	7.7638
KET-BP	0.81437	2.5011	5.6938
NDO-BP	0.80957	2.5332	5.8542
ESC-BP	0.82642	2.4186	5.7475
DBA-BP	0.82637	2.4190	5.7111
ALA-BP	0.82575	2.4233	5.2561
HHO-BP	0.79207	2.6471	6.0413
PSO-BP	0.80748	2.5471	5.7920
SMA-BP	0.83048	2.3901	5.3162
UFIA-BP	0.81040	2.5277	5.8381
MSKET-BP	0.86440	2.1377	5.0083

## Data Availability

The original contributions presented in this study are included in the article. Further inquiries can be directed to the corresponding author.
